# Direct Conversion of Syngas to Higher Alcohols via Tandem Integration of Fischer–Tropsch Synthesis and Reductive Hydroformylation

**DOI:** 10.1002/anie.202201004

**Published:** 2022-05-31

**Authors:** Kai Jeske, Thorsten Rösler, Maurice Belleflamme, Tania Rodenas, Nico Fischer, Michael Claeys, Walter Leitner, Andreas J. Vorholt, Gonzalo Prieto

**Affiliations:** ^1^ Department for Heterogeneous Catalysis Max-Planck-Institut für Kohlenforschung Kaiser-Wilhelm-Platz 1 45470 Mülheim an der Ruhr Germany; ^2^ Max Planck Institute for Chemical Energy Conversion Stiftstraße 34–36 45470 Mülheim an der Ruhr Germany; ^3^ ITQ Instituto de Tecnología Química Universitat Politècnica de València-Consejo Superior de Investigaciones Científicas (UPV-CSIC) Avenida de los Naranjos s/n 46022 Valencia Spain; ^4^ Catalysis Institute and DSI-NRF Centre of Excellence in Catalysis c✶change Department of Chemical Engineering University of Cape Town Cape Town, Rondebosch 7701 South Africa; ^5^ Institut für Technische und Makromolekulare Chemie RWTH Aachen Worringerweg 2 52074 Aachen Germany

**Keywords:** Cascade Reactions, Higher Oxygenates, Plasticizer Alcohols, Syngas Conversion, Tandem Catalysis

## Abstract

The selective conversion of syngas to higher alcohols is an attractive albeit elusive route in the quest for effective production of chemicals from alternative carbon resources. We report the tandem integration of solid cobalt Fischer–Tropsch and molecular hydroformylation catalysts in a one‐pot slurry‐phase process. Unprecedented selectivities (>50 wt %) to C_2+_ alcohols are achieved at CO conversion levels >70 %, alongside negligible CO_2_ side‐production. The efficient overall transformation is enabled by catalyst engineering, bridging gaps in operation temperature and intrinsic selectivity which have classically precluded integration of these reactions in a single conversion step. Swift capture of *1*‐olefin Fischer–Tropsch primary products by the molecular hydroformylation catalyst, presumably within the pores of the solid catalyst is key for high alcohol selectivity. The results underscore that controlled cooperation between solid aggregate and soluble molecular metal catalysts, which pertain to traditionally dichotomic realms of heterogeneous and homogeneous catalysis, is a promising blueprint toward selective conversion processes.

## Graphical abstract

Catalyst engineering enables the single‐pot integration of solid nanoparticulate and molecular cobalt catalysts in a slurry‐phase tandem Fischer–Tropsch synthesis/olefin reductive hydroformylation process for a direct and highly selective conversion of syngas (H_2_/CO) to higher alcohols. The new tandem process contributes to bridging the traditionally dissevered disciplines of heterogeneous and homogeneous catalysis.

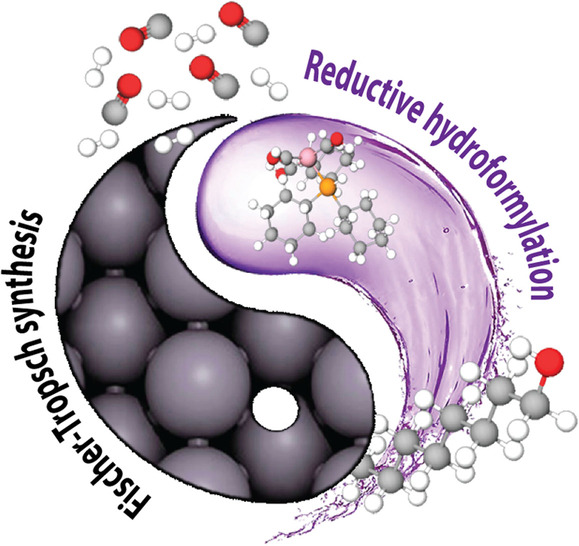

## Introduction

The direct conversion of synthesis gas (H_2_/CO) into value‐added chemicals is a highly attractive route to connect oil‐alternative carbon feedstocks, ranging from (unconventional) natural gas to lignocellulosic biomass and CO_2_, e.g. in the context “Power‐to‐X” concepts, to existing value chains.^[1]^ Higher (C_2+_) alcohols are sought products owing to their widespread applications as solvents, surface modifiers and precursors for ester plasticizers and fatty acid surfactants, among other commodities,^[2]^ while applications as high‐cetane soot‐inhibitors in (biogenic) diesel fuels have been explored recently.^[3]^ Catalytic addition of syngas (“hydroformylation”) to linear olefins from cracking (C_2–4_) or ethylene oligomerization (C_6+_) is a major industrial route to higher alcohols.^[4]^ The development of an alternative route towards technologies assembling the entire carbon skeleton in the product molecules from syngas remains a major challenge.

The catalytic conversion of syngas to higher alcohols has received a significant deal of research efforts.^[5]^ Already at the earliest development of the Fischer–Tropsch synthesis (FTS), alcohols were detected in varying shares within the hydrocarbon products, particularly in olefin recycling experiments. This observation was inspirational to Fischer's collaborator Otto Roelen for the development of the “oxo‐reaction” (hydroformylation, HF) of olefins to aldehyde/alcohol derivatives that marked the starting point for industrial‐scale homogeneous catalysis.^[6]^ Ever since, various families of catalysts have been developed for the direct conversion of syngas to higher alcohols.^[7]^ However, insufficient selectivity and yields have precluded industrial application hitherto.

Alkali‐modified Cu‐based methanol synthesis catalysts,^[8]^ oxide‐promoted rhodium catalysts,^[9]^ and alkalinized MoS_2_ catalysts^[10]^ deliver relatively short alcohol products, chiefly with up to four carbon atoms (C_4−_). Higher selectivities to mid‐chain (C_3_–C_10_) linear alcohols have been reported using CoCu bimetallic catalysts, designed as an attempt to combine the dissociative CO hydrogenation and C−C polymerization functionalities of the Co‐catalyzed FTS with the non‐dissociative CO activation intrinsic to the Cu‐catalyzed methanol synthesis.^[11]^ However, activity towards the water‐gas‐shift reaction (WGSR) typically results in increasingly high selectivities to CO_2_ at industrially relevant CO conversion levels.[^11b^, ^12^] Non‐dissociative CO insertion to produce higher alcohols has also been demonstrated for catalysts exhibiting Co‐Co_2_C interfaces, such as CoMn systems.^[13]^ Common to most of the catalyst families listed above is that, the side‐activity for WGSR, particularly at high CO conversion levels, sets limits to the overall carbon yield to higher alcohols and it is particularly undesired to valorize hydrogen‐rich syngas mixtures (H_2_ : CO≈2), such as those derived from natural gas reforming or biomass steam gasification.

Owing to the above limitations of direct conversion approaches, at present, the production of higher alcohols from syngas would inevitably rely on multi‐step processes comprising firstly the FTS of synthetic hydrocarbons enriched in linear olefins, followed by hydroformylation of the latter to the corresponding aldehyde derivatives with additional syngas, and finally aldehyde hydrogenation to alcohols, or reductive olefin hydroformylation (RHF) directly to alcohol end‐products.^[14]^ While attractive as a process intensification strategy, the tandem integration of the FTS and olefin (R)HF conversion steps into a one‐pot transformation has long been impeded by incompatibility in process parameters such as operating temperatures.^[15]^ Olefin‐producing FeC_*x*_‐based FTS catalysts operate at temperatures >513 K, at which the molecular HF catalysts are not stable.[^5a^, ^16^] In contrast, Co‐based FTS catalysts are effective at milder temperatures of 443–493 K, and essentially inactive to the WGSR, hence ideal to convert H_2_‐rich syngas.^[17]^ However, their high activity for secondary olefin hydrogenation leads to highly paraffinic products with low shares of liquid olefins, which are needed as educts for hydroformylation.

**Scheme 1:**
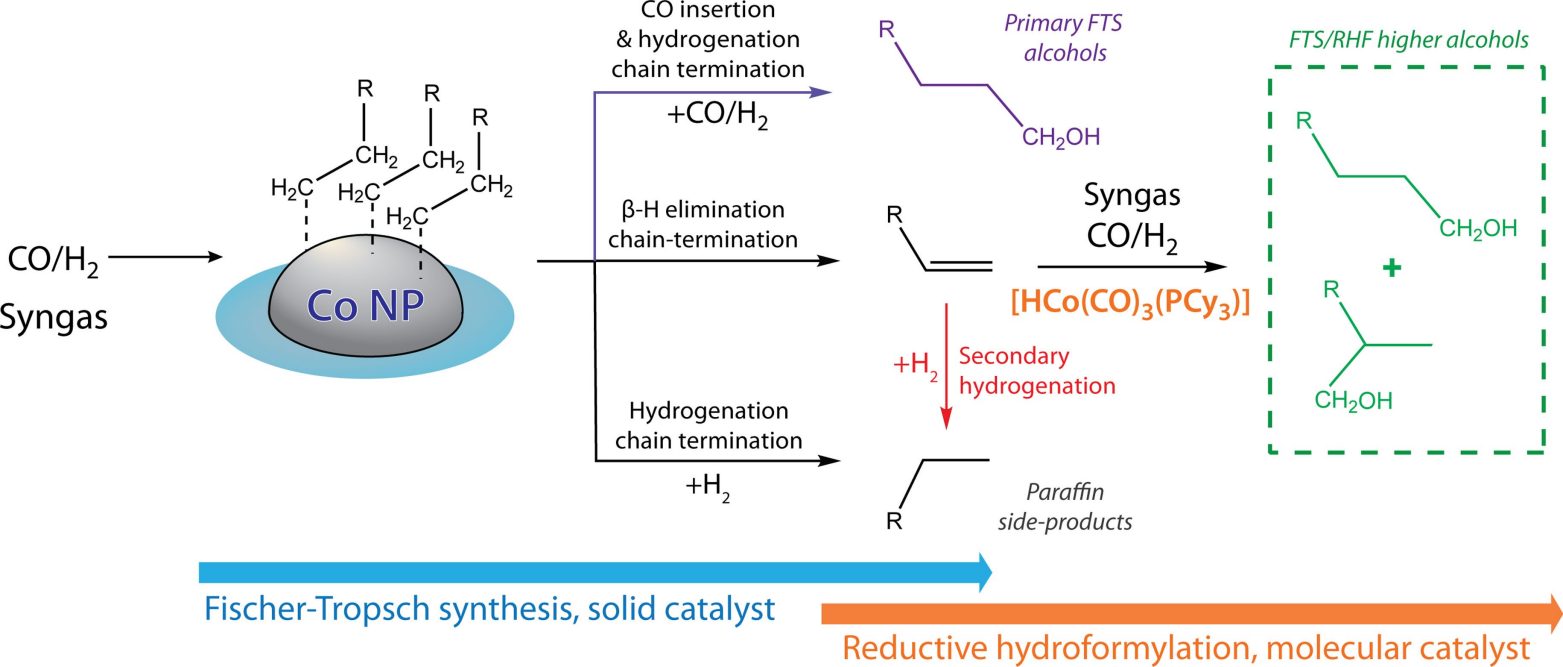
Production of higher alcohols from syngas via the integration of Fischer–Tropsch synthesis (FTS) and reductive hydroformylation (RHF) in a tandem process. The active sites of the solid catalyst are responsible for chain growth (number of C_*n*_ in R). The ratio between β‐H elimination and hydrogenation as chain termination steps determines the primary hydrocarbon selectivity, i.e. olefin‐to‐paraffin ratio. The activity of the molecular catalyst in olefin reductive hydroformylation relative to secondary olefin hydrogenation pathways determines the final alcohol selectivity. For completeness, the CO insertion chain‐termination pathway on the FTS catalyst is also indicated as a source for primary, strictly linear higher alcohols.

Motivated by our investigations on multimodally porous cobalt FTS catalysts, which deliver unconventionally high selectivities to higher olefins at mild temperatures (<493 K),^[18]^ and our investigations regarding the RHF of olefins,^[19]^ we herein show that these heterogeneously and homogeneously catalyzed reactions can be integrated in a tandem process (Scheme 1), enabling a direct and highly selective conversion of syngas to higher alcohols.

## Results and Discussion

As a surface polymerization reaction, the C_3+_ chain‐length distribution for FTS hydrocarbons is ideally described by a single parameter, i.e. the chain‐growth probability (α), according to an Anderson–Schulz–Flory (ASF) mathematical function.^[20]^ However, in supported metal FTS catalysts, secondary reactions, enhanced by the sluggish transport of primary FTS products through catalyst pores, are additionally determinant for the product pattern both in terms of hydrocarbon chain‐length as well as saturation degree.^[17]^ Hence, judicious adjustment of catalyst porosity, to control intrapore mass transport kinetics, serves as a handle on product selectivity. It has been recently shown by us that the design of cobalt‐based FTS catalysts with multimodal porosities enables the conversion of syngas into synthetic linear hydrocarbons with unconventionally high shares of C_3+_ olefins.[^18^, ^21^] Here, these previous findings have been further refined to develop a hierarchically porous CoRu/γ‐Al_2_O_3_ FTS catalyst, dually promoted with NaO_*x*_ and PrO_*x*_ basic oxides (NaPr‐CoRu/AOmM, where mM indicates the coexistence of meso‐ and macropores), which affords an extraordinarily high selectivity to C_3+_ *1*‐olefins.

**Figure 1:**
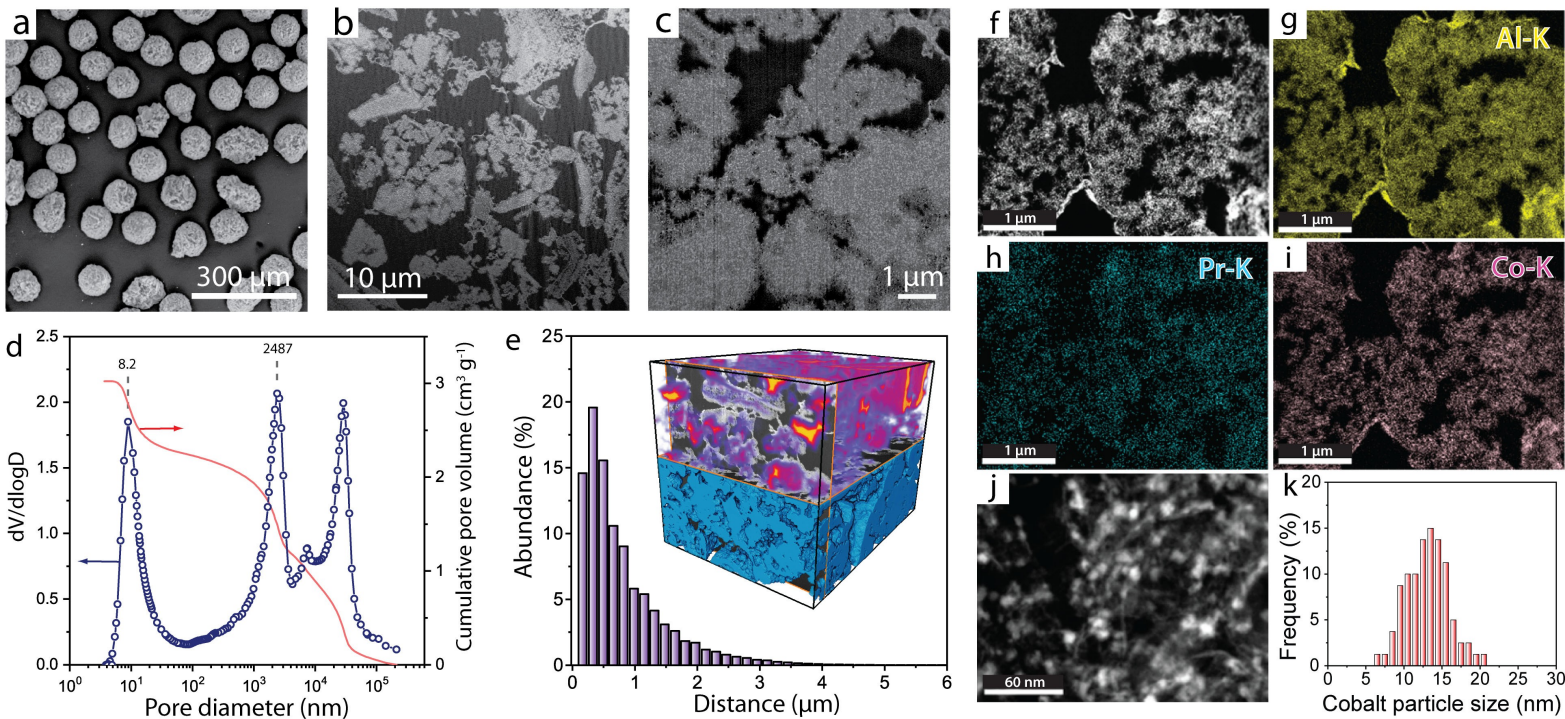
Structural characterization of an olefin‐selective cobalt Fischer–Tropsch catalyst (NaPr‐CoRu/AOmM). a) SEM image for catalyst microparticles. b), c) Cross‐sectional SEM images after Focused‐Ion‐Beam (FIB) milling of the resin‐embedded catalyst showing macropore opening cross‐sections (black regions) delimited by the γ‐Al_2_O_3_ backbone (light gray). d) Differential (blue scatter) and cumulative (red line) Hg intrusion pore size distributions. The contribution at >10^4^ nm corresponds to voids between the catalyst microparticles. e) Histogram for the maximum transport distance through mesopore regions to the nearest boundary with the network of macropores as derived from 3D image analysis of the FIB‐SEM tomogram. The inset shows the 3D contour plot for the Euclidean distance to nearest macropore (top half) overimposed to the reconstructed tomogram (bottom half), with mesoporous Al_2_O_3_ domains displayed in blue. f) C_S‐_HAADF‐STEM image and g)–i) EDS analysis maps recorded at the Al‐, Pr‐ and Co‐K emission lines, respectively, on ultramicrotomed catalyst cross sections. Darkest regions (lowest Z‐contrast) correspond to the resin‐filled macropore openings. j) High‐magnification C_S_‐HAADF‐STEM image showing cobalt nanoparticles (brightest speckles with higher Z‐contrast) confined to the network of γ‐Al_2_O_3_ sheet‐like nanocrystallites. k) Cobalt particle size histogram.

As shown in Figure 1 cross‐sectional scanning electron microscopy (SEM) after focused‐ion‐beam (FIB) milling revealed a very open porosity, with wide macropore openings delimited by a backbone of aggregated γ‐Al_2_O_3_ nanocrystals. Higher magnification imaging with high‐angle annular dark‐field scanning‐transmission electron microscopy (C_s_‐HAADF‐STEM) coupled to energy‐dispersive X‐ray spectroscopy showed cobalt nanoparticles (13.4±4.1 nm) confined to the mesoporous γ‐Al_2_O_3_ domains and a homogeneous mesoscale spatial distribution of the PrO_*x*_ promoter oxide. A markedly bimodal intraparticle porosity was ascertained by Hg intrusion porosimetry (Figure 1d). A set of mesopores (ca. 8 nm), which hosts the catalytically active cobalt nanoparticles, is complemented by a set of wider macropores accessed via openings with diameters centered at about 2.5 μm. As revealed with quantitative FIB‐SEM tomography analysis, the 3D maximum transport length across the metal‐loaded mesoporous domains to the boundary with the nearest macropore, which is relevant for pore residence time and secondary reactions of primary FTS products, averages 0.57±0.02 μm, i.e. it is reduced two orders of magnitude shorter compared to the catalyst microparticle diameter (Figure 1e). For reference purposes, a CoRu/AOm was synthesized by dispersing similarly sized metal nanoparticles on a standard γ‐Al_2_O_3_ carrier with identical surface area (Figure S1) but a strictly mesoporous architecture (Figure S2), which results in notably longer maximum mesopore transport distances, in the range of the microbeads radius (50 μm).

**Figure 2:**
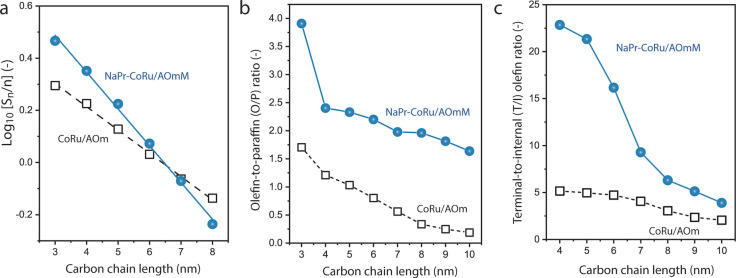
Higher olefin‐selective, fixed‐bed Fischer–Tropsch synthesis with cobalt catalysts. a) Linearized Anderson–Schulz–Flory hydrocarbon product distributions. b) Evolution of the olefin‐to‐paraffin molar ratio with hydrocarbon chain length in the C_3–10_ product range; and c) evolution of the terminal‐to‐internal molar ratio for olefin products in the C_4–10_ product range for a conventional mesoporous CoRu/γ‐Al_2_O_3_ (CoRu/AOm) and a dually promoted hierarchically porous NaO_x_, PrO_*x*_‐CoRu/γ‐Al_2_O_3_ (NaPr‐CoRu/AOmM) cobalt‐based Fischer–Tropsch catalysts. Reaction conditions: *T*=473 K, *P*=20 bar, H_2_ : CO=2, CO conversion=20±3%.

Under industrially relevant FTS gas‐solid operation conditions (*P*=20 bar, *T*=473 K, H_2_ : CO=2) NaPr‐CoRu/AOmM showed a lower metal‐specific reaction rate than the standard CoRu/AOm (Table S1), an effect which is associated to the dampening of the intrinsic hydrogenation activity caused by the presence of NaO_*x*_ and PrO_*x*_ oxide promoters on the surface of the cobalt nanoparticles.^[21]^ Analysis of the hydrocarbon distributions revealed no major differences in chain‐growth probability, which amounted in both cases to 0.78±0.01 (Figure 2a). However, the combination of the hierarchical porosity with the oxide surface promotion in NaPr‐CoRu/AOmM led to significant boosts in both the olefin‐to‐paraffin molar ratio for all hydrocarbon products in the C_3–10_ range (Figure 2b), as well as in the abundance of terminal isomers among olefin products (Figure 2c). These combined effects, which are ascribed to the inhibition of hydrogenation and isomerization secondary reactions for *1*‐olefin primary products, resulted in an almost threefold surge in the overall selectivity to C_3–10_ olefins, which reached 31.8 C% (Table S1). This is among the highest selectivities to higher *1*‐olefins ever reported for a cobalt‐based FTS catalyst.[^13b^, ^22^] Remarkably, this unusual selectivity pattern is achieved while maintaining a very low CO_2_ side‐production (0.9 C%), a performance pattern which is out of reach for traditional olefin‐productive FeC_x_ FTS catalysts. The high selectivity to higher *1*‐olefins at mild reaction temperature (473 K) makes this catalyst an excellent candidate for a one‐pot integration with olefin RHF catalysis.

Molecular cobalt‐based hydroformylation catalysts are known since the earliest times of the process, and still applied industrially. Compared to widespread Rh‐based catalysts, cobalt carbonyl compounds stabilized by organo‐phosphine ligands are known to be highly active for the direct olefin RHF to alcohols, while they remain functional at temperatures *T*>393 K,^[23]^ which are important assets in the context of herein investigated tandem FTS/RHF process. Particularly trialkylphosphine ligands are stronger *σ*‐donors than arylphosphine analogues, and assist the hydrogenation activity to alcohol end‐products.^[23a]^ Under reaction conditions representative for the FTS (468 K, *P*=120 bar, H_2_ : CO=2), a combination of Co_2_(CO)_8_ with tricyclohexylphosphine (PCy_3_) ligand converted *1*‐octene, a model for mid‐chain FTS *1*‐olefin products, with very high selectivity to C_9_ alcohols (93 %) and low side‐production of the full hydrogenation product *n*‐octane (7 %) even at full olefin conversion (Table S2, entry 2.1). Phosphorus Nuclear‐Magnetic‐Resonance (^31^P‐NMR) spectroscopy proved the presence of two dimeric organometallic species [Co_2_(CO)_6_(PCy_3_)_2_] and [Co_2_(CO)_7_(PCy_3_)] in the reaction medium. These species have been proposed to be precursors in equilibrium with the catalytically active monomeric species [HCo(CO)_3_(PCy_3_)],^[24]^ whose formation was also ascertained by ^1^H‐NMR (Figures S3–S5). The CO partial pressure (*P*_CO_) showed a substantial effect on performance. Decreasing *P*_CO_ below 30 bar led to a significant decrease in RHF activity, and thus alcohol selectivity (by 83 % at *P*_CO_=10 bar) in favor of paraffin and olefin isomer side‐products (Table S2, 2.4), suggesting that a critical *P*_CO_ (≈30 bar) is required to sustain a maximum concentration of RHF‐active Co carbonyl species in solution. The ligand‐to‐metal ratio (L : M), defined as the P : Co molar ratio, showed a marked effect on product (regio)selectivity (Table S3). RHF of *1*‐olefin substrates leads to either linear (*anti*‐Markovnikov) or branched (Markovnikov) alcohol products (Scheme 1). Branched *iso*‐alcohols can also result from RHF of internal olefin isomers formed by secondary double‐bond isomerization prior to hydroformylation (Figure S6). Increasing L : M resulted in a steep increase in the linear‐to‐branched (*n* : *iso*) alcohol ratio, however, at the expense of an enhanced olefin hydrogenation (Figure S3). Accordingly, a P : Co of 1 : 1, i.e. the stoichiometric value in the [HCo(CO)_3_(PCy_3_)] complex, was selected to maximize alcohol selectivity.

Tandem FTS/RHF catalytic tests were performed in a slurry‐phase batch reactor, using *2*‐methyl pentane as solvent and a syngas feed with H_2_ : CO=2 (full experimental details in the Supporting Information). The molecular RHF catalyst was found to develop during an induction period when the Co_2_CO_8_ metal precursor and the organo‐phosphine ligand were directly blended into the reaction medium. Alternative tests preceded by a catalyst *pre‐forming* step, where metal carbonyl and ligand precursors were heated in the reaction solvent under syngas pressure prior to the incorporation of the solid FTS catalyst, led to essentially the same results indicating that in situ RHF catalyst formation in presence of the suspended solid catalyst is equally efficient (Figure S7). ^31^P‐NMR showed the existence of the [HCo(CO)_3_(PCy_3_)] complex in solution in either case. (Figure S8).

First, the NaPr‐CoRu/AOmM FTS catalyst was combined in a single reaction pot with the in situ formed [HCo(CO)_3_(PCy_3_)] RHF catalyst. The ratio between the two catalysts, expressed on a cobalt molar basis, was Co_FTS_/Co_RHF_=170 : 18. A CO conversion of 35 % was achieved after 24 h at 473 K (Table 1, entry 1.1), whilst the total selectivity to alcohols reached 37.0 C%. The alcohol products followed an ASF distribution, with a chain‐growth probability of α_ROH_=0.58, indicating that their hydrocarbon molecular backbone originated from the conversion of syngas through the FTS (Figure S9). Strikingly, essentially no olefins were detected in the products (<0.2 C%), suggesting their quantitative secondary conversion via RHF and/or hydrogenation paths into the corresponding C_*n*+1_ alcohol or C_*n*_ paraffin products, respectively. The latter were the major reaction products and showed a slightly higher α_HC_=0.71. Moreover, methane and CO_2_ selectivities remained low at 10.3 and 1.8 C%, respectively, i.e. levels comparable to those of a standard cobalt‐catalyzed FTS.^[25]^

**Table 1:** CO conversion (X_CO_) and product selectivities (molar carbon base) for slurry‐phase tandem catalysis syngas conversion experiments with different combinations of cobalt solid Fischer–Tropsch synthesis and molecular reductive hydroformylation catalysts, as well as component exclusion reference experiments. Reaction conditions: *T*=473 K, *P*=120 bar (initial, measured at RT), stirring rate=700 rpm, syngas feed H_2_ : CO=2.0, 2‐methyl pentane as solvent. For tests integrating both FTS and RHF catalysts, catalyst ratio *n*(Co_FTS_)/*n*(Co_RHF_)=170/18 (entry 1.1) or 170/70 (entry 1.4, 1.6–1.9), reaction time *t*=24 h. For tests where both components forming the RHF catalyst were present the ligand‐to‐metal (L : M) ratio, i.e. P/Co=1 : 1 (at/at). Ligand abbreviations: [1] PCy_3_: tricyclohexyl phosphine. [2] PPh_3_: triphenyl phosphine. [3] P(*n*‐Bu)_3_: tri‐*n*‐butyl phosphine.

Entry	Catalyst		X_CO_	*S*(CO_2_)	*S*(CH_4_)	*S*_C5+_	α_HC_^[a]^	*S*_olef._	*S*_ROH_	*S*_C5+ ROH_	ROH^[b]^	α_ROH_^[c]^
	FTS	RHF	[%]	[C%]	[C%]	[C%]	[C%]	[C%]	[C%]	[C%]	*n* : *iso*	[−]
1.1	NaPr‐CoRu/AOmM	Co_2_CO_8_ + PCy_3_ ^[1]^	35.1	1.8	10.3	66.9	0.71	0.1	37.0	25.4	4.2	0.58
1.2	NaPr‐CoRu/AOmM	–	36.1	1.5	6.3	64.9	0.66	0.2	25.5	12.6	2.2	0.54
1.3	NaPr‐CoRu/AOmM	PCy_3_	21.8	1.1	9.5	55.4	0.63	0.1	33.6	15.8	6.4	0.44
1.4	NaPr‐CoRu/AOmM	Co_2_CO_8_	41.8	1.6	9.4	50.6	0.64	0.8	19.5	9.2	2.1	0.54
1.5	–	Co_2_CO_8_+PCy_3_	0.2	>60	>20	<0.1	n.a.	0.0	0.0	0.0	n.a.	n.a.
1.6	NaPr‐CoRu/AOmM	Co_2_CO_8_+PPh_3_ ^[2]^	28.8	2.3	8.6	55.3	0.70	<0.1	47.7	25.4	3.1	0.57
1.7	NaPr‐CoRu/AOmM	Co_2_CO_8_+(P(*n*‐Bu)_3_)^[3]^	26.8	4.9	8.9	53.8	0.69	<0.1	53.9	30.5	4.6	0.53
1.8	NaPr‐CoRu/AOmM	Co_2_CO_8_+PCy_3_	34.0	2.2	6.9	59.1	0.71	<0.1	53.7	22.7	5.8	0.61
1.9	CoRu/AOm	Co_2_CO_8_+PCy_3_	24.5	1.1	9.4	74.5	0.84	<0.1	32.7	23.3	4.4	0.66

[a] Hydrocarbon chain‐growth probability. [b] Alcohol linear‐to‐branched molar ratio. [c] Alcohol chain‐growth probability.

Control experiments were conducted to elucidate the role of different species (Table 1, 1.2–1.5). Adding the FTS catalyst NaPr‐CoRu/AOmM as the sole catalyst resulted in a similar CO conversion after 24 h (36 %, Table 1, 1.2), confirming that this catalyst is responsible for the primary conversion of syngas in the tandem process. The alcohol selectivity was 25.5 C%, i.e. significantly lower than that achieved in the tandem under identical operation settings, which verified the important role of the RHF molecular catalyst for an effective oxo‐functionalization of FTS products in situ. It was, however, higher than for gas‐phase FTS experiments with the same catalyst (Table S1). Moreover, essentially no olefins (0.2 C%) were detected as products, also in marked contrast to gas‐solid FTS tests (Figure S10). These observations suggested that a certain olefin hydroformylation activity might be at play with the solid FTS catalyst in slurry‐phase, possibly driven by cobalt carbonyl compounds which might develop on the surface of the metal nanoparticles or leach into solution.^[26]^ Indeed, a minor albeit measurable concentration of cobalt (7 ppm) was detected in the reaction liquors by inductively‐coupled plasma mass‐spectrometry (ICP‐MS) (Table S4). The premise that these species are responsible for a certain background RHF activity is further supported by the fact that the produced alcohols are not exclusively linear, as observed in gas‐solid FTS tests (Figure S11) and expected if CO‐insertion chain termination in a FTS mechanism is their sole genesis (Scheme 1).^[27]^ Next to RHF, secondary hydrogenation due to the extended residence time of olefin primary FTS products in contact with the metallic centers of the solid catalyst in slurry‐phase tests might have additionally contributed to olefin depletion.

The addition of the PCy_3_ alongside the solid FTS catalyst lowered CO conversion (22 %) but already enhanced the selectivity to alcohols to 33.6 % (Table 1, 1.3). This suggests that formation of molecular RHF‐active species by partial leaching of cobalt species from the surface of the supported metal nanoparticles was enhanced by the excess phosphine ligand. Supporting this proposal, a higher cobalt concentration was detected in solution (51 ppm, Table S4) and ^31^P‐NMR confirmed the presence of [HCo(CO)_3_(PCy_3_)] in solution, despite the exclusion of a specific metal precursor for this molecular catalyst (Figure S12). The high *n* : *iso* ratio observed under these conditions is in line with the excess phosphine in solution. A control experiment combining the FTS catalyst only with the Co_2_CO_8_ molecular precursor (in the absence of organic ligand), led to much lower alcohol selectivity (19.5 C%) (Table 1, 1.4). Adding the [HCo(CO)_3_(PCy_3_)] complex as a single catalyst, resulted in negligible syngas conversion (Table 1, 1.5). Jointly, these results furnish strong evidence for the need to integrate both the solid FTS and the molecular RHF catalysts to efficiently produce higher alcohols.

Next to PCy_3_, the application of alternative tri‐alkyl and tri‐aryl phosphine ligands to stabilize the molecular RHF catalyst in the tandem reaction promoted also high alcohol selectivities, pointing to the general validity of the syngas conversion concept (Table 1, 1.6 and 1.7). However, selectivities to CO_2_ >2 % in those cases, indicated a certain exacerbation of the WGSR with these weaker *σ*‐donor organo‐phosphine ligands. Moreover, replacement of NaPr‐CoRu/AOmM in the tandem (Table 1, 1.8) by a conventional CoRu/AOm FTS catalyst (Table 1, 1.9) led to a decrease in the total alcohol selectivity from >50 % to 32.7 %. This underscores the significance of engineering the FTS catalyst towards unconventionally high selectivities to higher terminal olefins, which are essential educts for the RHF reaction.

**Figure 3:**
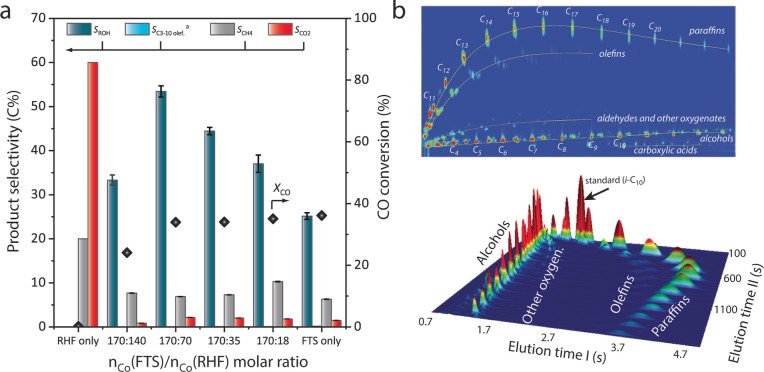
Direct syngas conversion to higher alcohols via the tandem Fischer–Tropsch synthesis/reductive olefin hydroformylation syngas conversion process. a) Product selectivity (left *y*‐axis) and CO conversion (right *y*‐axis) for slurry‐phase tandem FTS/RHF experiments as a function of the *n*(Co_FTS_)/*n*(Co_HyFo_) catalyst ratios. FTS catalyst: NaPr‐CoRu/AOmM; RHF catalyst: Co_2_CO_8_+P(Cy)_3_ (L : M=1.0 (P/Co (at/at)). The extreme cases, i.e. the FTS and RHF catalysts tested independently, are also included for reference. Reaction conditions: *T*=473 K, *P*=120 bar (initial, measured at RT), stirring rate 700 rpm, syngas feed H_2_:CO=2,2‐methyl pentane as solvent. [a] C_3–10_ olefin selectivity ≤0.2 C% in all cases. The test with the RHF catalyst alone led to a too low (0.2 %) CO conversion, at which the C balance closed only at 80 %. b) Representative two‐dimensional gas chromatogram for the liquid products in a slurry‐phase FTS/RHF tandem reaction test under optimized reaction conditions.

The effect of the FTS/RHF catalyst ratio on the tandem conversion performance was assessed for the combination of NaPr‐CoRu/AOmM and [HCo(CO)_3_(PCy_3_)] catalysts. As shown in Figure 3a, progressively increasing the relative amount of the molecular RHF catalyst from Co_FTS_/Co_RHF_=170 : 18 to 170 : 70 resulted in a continuous increase in the selectivity to alcohols at essentially constant CO conversion (30±6 %), indicating a higher capacity to oxo‐functionalize primary FTS olefins as they formed. At a Co_FTS_/Co_RHF_ ratio of 170 : 70 alcohols were the major reaction product reaching a selectivity of 53.7 C% (or 59.6 wt %) at a CO conversion level of 34 %. The corresponding selectivity to C_5+_ alcohols was 22.7 C%. Moreover, as a result of the moderate alcohol chain‐growth probability of 0.61, >79 C% of these alcohol products spanned in the chain‐length regime of C_4‐13_, i.e. covering the entire range of industrially relevant plasticizer alcohols. 2D gas chromatography identified other oxygenate products, i.e. aldehydes and carboxylic acids, though only at trace levels (Figure 3b). Further decreasing Co_FTS_/Co_RHF_ to 170 : 140 resulted in a drop in both CO conversion and alcohol selectivity. Therefore, striking a balance in the abundance of active sites for both reactions is essential for optimal performance. Following a tandem FTS/RHF test, the FTS catalyst showed no detectable nanostructure changes (Figure S13), whilst neither Na nor Pr were detected by ICP‐MS/ICP‐OES in the reaction liquors. The catalyst could be recovered and reused in a second reaction run without signs of deactivation (Figure S14).

The selectivity to higher alcohols achieved with the tandem process (e.g. Table 1, entry 1.8) was slightly higher (by ca. 15 %) than the lumped selectivity to alcohols and olefins delivered by the FTS catalyst independently (Table S1, entry 1.2). This suggested fast trapping of FTS primary olefin products by the RHF molecular catalyst, preventing to a certain extent olefin re‐adsorption on Co nanoparticles and thus secondary hydrogenation to paraffins. Insights into the operation mode of the tandem system were gathered via analysis of alcohol regioselectivity. While primary FTS alcohol products are exclusively linear (see Figures EM3 and S11), mixtures of *n*‐ and *iso*‐alcohols result from secondary RHF of FTS olefins in the tandem system (Scheme 1). Linear‐to‐branched‐alcohol (*n* : *iso*) ratios were systematically higher for the tandem system (>2) compared to the 1.3–1.6 ratio observed with the standalone [HCo(CO)_3_(PCy_3_)] RHF catalyst on *1*‐olefin model substrates (Table S2). A priori, such excess in linear alcohols in the tandem process cannot be accounted for solely on the basis of the contribution of primary FTS *n*‐alcohols. The additional *n*‐alcohol excess could originate from an intrinsic regioselectivity of the RHF catalyst or if this molecular catalyst could intercept α‐C‐bonded growing hydrocarbon chains from the surface of the Co nanoparticles on the FTS catalyst, prior to their desorption, e.g. via an alkyl‐shift mechanism (Figure S15). To shed light on this matter, the fate of *1*‐olefin reactants in the tandem reaction was independently assessed by co‐feeding *5*‐methyl‐*1*‐hexene, a *1*‐olefin with a branched hydrocarbon backbone which cannot be produced from syngas via the FTS, as a tracer substrate (Table S5). The *n* : *iso* for C_8_ alcohol products derived from the exogenous *1*‐olefin was 2.3, whereas that for alcohols with no methyl substituents farther away from the β‐C atom, i.e. those which evolve either directly from the FTS or as a result of subsequent RHF of FTS olefins, amounted to 3.0. On the one hand, these results emphasize that the RHF catalyst inherently favors linear products under the tandem process conditions delivering higher *n* : *iso* ratios than when applied individually on model *1*‐olefin substrates. On the other hand, the minor differences in isomer distribution for alcohol products derived from either tracer (exogeneous) or endogenic olefins can be explained by considering the contribution from FTS primary *n*‐alcohol products to the latter, discarding different mechanistic RHF paths for tracer and FTS *1*‐olefins. These results furnish evidence for a sequential conversion wherein both catalysts operate independently, without major cross‐interaction. Olefin primary products which desorbed from the FTS catalyst react further on the molecular RHF catalyst in the liquid phase.

To elucidate the exchange of intermediate olefin products in the tandem reaction, we set to assess the impact of spacing and transport between the active centers of the two catalysts on performance. As a means to spatially separate the active sites of both catalysts and prevent infiltration of the RHF catalyst within the pores of the FTS catalyst, the former was heterogenized by using polymer‐tethered organo‐phosphine ligands (see methods in the Supporting Information). The latter polymeric catalyst showed a RHF performance remarkably similar to that of the [HCo(CO)_3_(PCy_3_)] molecular counterpart (Figure S16). However, its application in the tandem process, i.e. restricting the RHF reaction to those olefins which have egressed from the particles of the solid FTS catalyst, lowered alcohol selectivity to less than half of that attained with the *free* molecular catalyst (Figure S16). This finding emphasizes the importance of close spatial proximity between the sites of the two catalysts to achieve high alcohol selectivity. In an additional experiment, a supercritical reaction medium was developed by replacing 2‐methylpentane for 2‐methylbutane (*T*_c_=461 K, *P*_c_=33.8 bar)^[28]^ as the reaction's solvent. Despite the enhancement of molecular transport coefficients expected in the supercritical medium,^[29]^ no increase in time‐yield or alcohol selectivity was observed (Figure S17), suggesting that the transport of olefin intermediate products between the sites of both catalysts is already sufficiently fast, *viz*. kinetically irrelevant, under conventional slurry‐phase conditions. In conjunction, these results demonstrate that the consecutive FTS and RHF reactions are mediated by a swift transport of olefin intermediate products between the sites of the FTS solid catalyst and the RHF molecular catalyst, likely within the porosity of the former.

**Figure 4:**
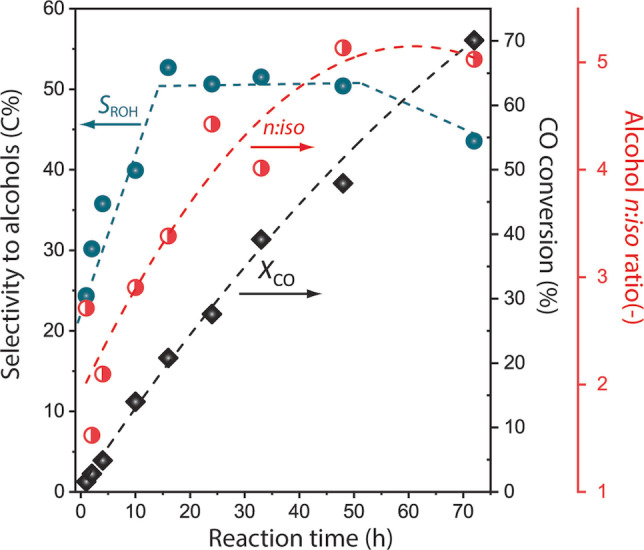
Time‐resolved evolution of CO conversion and alcohol product (regio)selectivity during the slurry‐phase tandem Fischer–Tropsch synthesis/reductive olefin hydroformylation syngas conversion process. FTS catalyst: NaPr‐CoRu/AOmM; RHF catalyst: Co_2_CO_8_+P(Cy)_3_ (L : M=1.0 (P/Co)). Reaction conditions: *T*=473 K, *P*=120 bar (initial, measured at RT), stirring rate 700 rpm, syngas feed H_2_:CO=2,2‐methyl pentane as solvent, catalyst ratio *n*(Co_FT_)/*n*(Co_HyFo_)=170/70. Dotted lines are included as guides to the eye.

Time‐resolved monitoring of the bifunctional process revealed a marked increment in alcohol selectivity with reaction time up to a CO conversion level of ca. 20 %. Very remarkably, total alcohol selectivities higher than 45 C% (or 50 wt %) and C_5+_ alcohol selectivities 27–32 C%, could be sustained up to CO conversion levels above 70 % (Figure 4, Table S6). Besides, in parallel to alcohol selectivity, a steep increase in the alcohol *n* : *iso* ratio was observed (from ca. 1.5 to >5.0) on increasing CO conversion up to ca. 50 %, which was not observed in the absence of the RHF catalyst and it is thus exclusive of the tandem system (Figure S18). These results prove the functionality of the catalyst system in the presence of those high water contents associated to high CO conversion levels. Moreover, a performance comparison to the state‐of‐the‐art (Figure S19) underscores that the combination of high selectivity to higher alcohols and very low WGSR activity, even at high CO conversion levels, unlocks unprecedented yields for the production of higher alcohols from syngas.

## Conclusion

An engineered cobalt Fischer–Tropsch catalyst with unconventionally high selectivity to higher olefins at mild reaction temperatures can be integrated in tandem with thermostable cobalt reductive hydroformylation catalysts to realize a slurry‐phase direct conversion of syngas to higher alcohols. Excellent selectivities to C_2+_ alcohols are achieved, even at high CO conversion levels, with alcohol chain‐lengths extending over the entire plasticizer range, remarkably, alongside minute CO_2_ side‐production. Given that technically uncomplicated approaches exist for the recovery and recycling of molecular Co‐based RHF catalysts,^[30]^ our results set excellent prospects for an efficient continuous process for the direct production of higher alcohols from syngas.

## Conflict of interest

The authors declare that a patent application has been filed on the process described herein, on which K.J., T. Rösler, M.B., W.L., A. J. V., and G.P are inventors.

1

## Supporting information

As a service to our authors and readers, this journal provides supporting information supplied by the authors. Such materials are peer reviewed and may be re‐organized for online delivery, but are not copy‐edited or typeset. Technical support issues arising from supporting information (other than missing files) should be addressed to the authors.

Supporting Information

## Data Availability

The data that support the findings of this study are available in the Supporting Information of this article.
