# Supplementary material for: Direct Conversion of Syngas to Higher Alcohols via Tandem Integration of Fischer–Tropsch Synthesis and Reductive Hydroformylation
Source: Angew Chem Int Ed Engl. 2022 May 31;61(31):e202201004. doi: 10.1002/anie.202201004 (PMC9400900; doi:10.1002/anie.202201004)
Supplement: Supplementary file 1 — Supporting Information [file ANIE-61-0-s001.pdf]

Angewandte Chemie
Eine Zeitschrift der Gesellschaft Deutscher Chemiker

Supporting Information

Direct Conversion of Syngas to Higher Alcohols via Tandem Integration of Fischer–Tropsch Synthesis and Reductive Hydroformylation

K. Jeske, T. Rösler, M. Belleflamme, T. Rodenas, N. Fischer, M. Claeys, W. Leitner, A. J. Vorholt*, G. Prieto**

Supporting Information

Direct Conversion of Syngas to Higher Alcohols via Tandem Integration of Fischer-Tropsch Synthesis and Reductive Hydroformylation

Kai Jeske[a,‡], Thorsten Rösler[b,‡], Maurice Belleflamme[b], Tania Rodenas[c], Nico Fischer[d], Michael Claeys[d], Walter Leitner*[b,e], Andreas J. Vorholt*[b] and Gonzalo Prieto*[a,c]

[a] K. Jeske, Dr. G. Prieto
Department for Heterogeneous Catalysis
Max-Planck-Institut für Kohlenforschung
Kaiser-Wilhelm-Platz 1, 45470 Mülheim an der Ruhr, Germany
E-mail: prieto@mpi-muelheim.mpg.de

[b] T. Rösler, M. Belleflamme, Prof. Dr. W. Leitner, PD Dr. A. J. Vorholt
Molecular Catalysis
Max Planck Institute for Chemical Energy Conversion
Stiftstraße 34-36, 45470 Mülheim an der Ruhr, Germany
Email: walter.leitner@cec.mpg.de; andreas-j.vorholt@cec.mpg.de

[c] Dr. T. Rodenas, Dr. G. Prieto
ITQ Instituto de Tecnología Química, Universitat Politècnica de València-Consejo Superior de Investigaciones Científicas (UPV-CSIC)
Avenida de los Naranjos s/n, 46022 Valencia, Spain.
Email: prieto@itq.upv.es

[d] Dr. N. Fischer, Prof. M. Claeys
Catalysis Institute and DSI-NRF Centre of Excellence in Catalysis c∗change, Department of Chemical Engineering, University of Cape Town
Rondebosch 7701, South Africa

[e] Prof. Dr. W. Leitner
Institut für Technische und Makromolekulare Chemie
RWTH Aachen University
Worringerweg 2, 52074 Aachen, Germany

<u>Table of contents</u> *Page*

1. Experimental methods S3
Figure EM1 ………………………………………….. S5
Figure EM2 ………………………………………….. S12
Figure EM3 ………………………………………….. S13
Table EM1 ………………………………………….. S17
Figure EM4 ………………………………………….. S18
Figure EM5 ………………………………………….. S19

2. Supporting Figures S20
Figure S1………………………………………………… S20
Figure S2 ……………………………………………….. S21
Figure S3 ……………………………………………….. S22
Figure S4 ……………………………………………….. S23
Figure S5 ……………………………………………….. S24
Figure S6 ……………………………………………….. S25
Figure S7 ……………………………………………….. S26
Figure S8 ……………………………………………….. S27
Figure S9 ……………………………………………….. S28
Figure S10 …………………………………………….. S29
Figure S11 …………………………………………….. S30
Figure S12 …………………………………………….. S31
Figure S13 …………………………………………….. S32
Figure S14 …………………………………………….. S33
Figure S15 …………………………………………….. S34
Figure S16 …………………………………………….. S35
Figure S17……………………………………………….. S36
Figure S18……………………………………………….. S37
Figure S19……………………………………………….. S38

3. Supporting Tables S39
Table S1 ………………………………………………….. S39
Table S2 ………………………………………………….. S40
Table S3 ………………………………………………… S41
Table S4 ………………………………………………… S42
Table S5 ………………………………………………… S43
Table S6 ………………………………………………… S44

4. References S45

1. Experimental methods

1.1. Catalyst synthesis

1.1.1. Synthesis of Co-based FTS catalysts

Supported cobalt-based catalysts were prepared by incipient wetness impregnation of porous γ-Al_2O_3 support materials. Mesoporous γ-Al_2O_3 (denoted AOm) and meso-macroporous γ-Al_2O_3 (denoted AOmM) were obtained via thermal dehydration of Catapal (Sasol Materials) and Versal (Honeywell UOP) microparticulate pseudo-boehmite precursors at 823 K (2 K min^{-1} heating rate) for 5 h in a muffle oven under stagnant air atmosphere. The resulting γ-Al_2O_3 supports were first dried under dynamic vacuum (423 K) for 2 hours prior to impregnation under static vacuum with a stock aqueous solution containing $Co(NO_3)_2{\cdot}6H_2O$ (1.5 M, Sigma-Aldrich, ≥98%, CAS: 10026-22-9) and ruthenium (III) nitrosyl nitrate (in dilute nitric acid, Sigma-Aldrich, CAS: 34513-98-9). To prepare this stock precursor solution, metal nitrates were dissolved in those amounts required to achieve an atomic ratio of Ru/Co=0.007, and the solution was further acidified with 0.25 vol% HNO_3 (69-70 vol.% in H_2O, J.T. Baker, ≥99%, CAS: 7697-37-2). The volume of said solution applied in the incipient wetness impregnation was equivalent to 90% of the total mesopore volume of the support material as determined by N_2 physisorption. Following impregnation, the solid was dried in a tubular reactor at 343 K under a vertical downward Ar flow (200 cm^3 g_{cat}^{-1} min^{-1}, Air Liquide, 99.999%, CAS: 7440-37-1) for 10 hours and the nitrate precursors further decomposed at 623 K for 4 h under Ar flow (heating rate of 1 K min^{-1} from RT). To adjust the total metal loading in the catalysts, five impregnation/calcination cycles were performed in the case of AOm, while two cycles sufficed in the case of AOmM. After each impregnation step, the total mesopore volume was corrected by the volume of the metal species previously deposited in the pores, assuming a density of 6.1 cm^3 g^{-1} (Co_3O_4).

1.1.2. Synthesis of promoted Co-based FTS catalysts

The required amount of the unpromoted CoRu/γ-Al_2O_3 catalyst (in its as-calcined oxidic form) was dried under dynamic vacuum (423 K) for 2 hours, and then impregnated with an aqueous solution of the nitrate precursors of the oxide promoters in 0.5 M HNO_3 (22.2 cm^3 of solution per unit cm^3 of solid mesopore volume). The concentration of the promoters nitrate precursor in the impregnating solution was adjusted to achieve a preset surface-specific content of 0.2 Na_{at} nm^{-2} and 0.8 Pr_{at} nm^{-2}. Given the specific support BET surface area of 293 m^2 g_{Al2O3}^{-1}, these translated into 11.3 mg of $NaNO_3$ (Sigma-Aldrich, >99%, CAS: 7631-99-4) and 231.1 mg

of $Pr(NO_3)_3 \cdot 6H_2O$ (Sigma-Aldrich, 99.9%, CAS: 15878-77-0) per gram of solid. After having slurried the solid in the impregnation solution, the water solvent was removed in a rotary evaporator at 323 K and the solid was subsequently transferred to a tubular reactor and calcined at 623 K for 4 h under vertical downward synthetic air flow (Air Liquide, 20.5 ± 0.5 mol% O_2 in N_2, 99.999%, CAS: 132259-10-0) using a heating rate of 1 K min^{-1} from RT and a gas flow of 200 cm^3 g_{solid}^{-1} min^{-1}.

1.2. Physicochemical characterization methods

1.2.1. N_2 physisorption porosimetry

N_2 physisorption isotherms of porous solids were recorded at 77 K using a Micromeritics 3Flex V4.04. Prior to the measurements, samples were dried *in situ* at 523 K under vacuum (10^{-3} mbar) for 12 h (10 K min^{-1}). The Brunauer-Emmett-Teller (BET) method was used to determine surface areas from the 0.05-0.30 P/P_0 regime of the recorded isotherms. Pore volumes were determined from the adsorbed N_2 volume detected at equilibrium point P/P_0 of 0.95 in the adsorption branch of the isotherm. Pore size distributions were determined using the Barrett-Joyner-Halenda (BJH) method applied to the desorption branch of the isotherm. The average mesopore diameter was defined as the peaking value of the pore size distribution.

1.2.2. Hg intrusion porosimetry

Mercury intrusion porosimetry experiments were performed in a Micromeritics AutoPore IV 951 apparatus. 80-150 mg of the solid sample (0.08-0.10 mm sieve fraction) were dried at 383 K for 72 h before the measurement. The intrusion-extrusion isotherms were recorded at room temperature in the pressure range of $6.9 \cdot 10^4$-$4.1 \cdot 10^4$ Pa with an equilibration rate of 0.1 μL g^{-1} s^{-1}. For the determination of pore diameter and volume, a geometrical pore model was considered, with a Hg density of 13.55 g cm^{-3} and a contact angle of 141 degree.

1.2.3. H_2-Temperature-programmed reduction

Hydrogen temperature-programmed reduction (H_2-TPR) experiments were conducted to assess the reducibility of metal species in supported FTS catalysts and select the temperature for the H_2-reduction treatment applied prior to catalysis to render the metal in its FTS-active metallic state. Experiments were performed in a Micromeritics Autochem 2910 device. About 45 mg of sample (0.08-0.10 mm sieve fraction) were initially

flushed with Ar flow (50 cm^3 min^{-1}) at room temperature for 30 min, then the gas was switched to 10 vol% H_2 in Ar and the temperature increased up to 1123 K at a heating rate of 10 K min^{-1}. A downstream acetone/dry ice trap, located downstream of the U-shaped tubular reactor, was used to retain the water generated during the reduction. The H_2 consumption rate was monitored by a thermal conductivity detector (TCD) previously calibrated via the injection of known volumes of hydrogen using a gas syringe. The cobalt loading in the catalysts was determined from the total hydrogen consumption assuming all cobalt to be present as Co_3O_4 in the starting calcined catalysts (as confirmed with powder X-ray diffraction experiments, not shown). Therefore a reduction stoichiometric H_2:Co molar ratio of 4/3 was applied. The hydrogen consumption associated to the reduction of the Ru promoter was considered negligible. Figure EM1 shows the H_2-TPR profiles registered for CoRu/AOm and NaPr-CoRu/AOmM Fischer-Tropsch synthesis catalysts. Reduction events peaking at 480-540 K and 650-660 K, respectively, were observed, corresponding to the two-step reduction of Co_3O_4 to metallic Co. In view of these reduction profiles, a reduction temperature of 673 K was selected to activate the FTS catalysts in this study prior to catalysis testing.

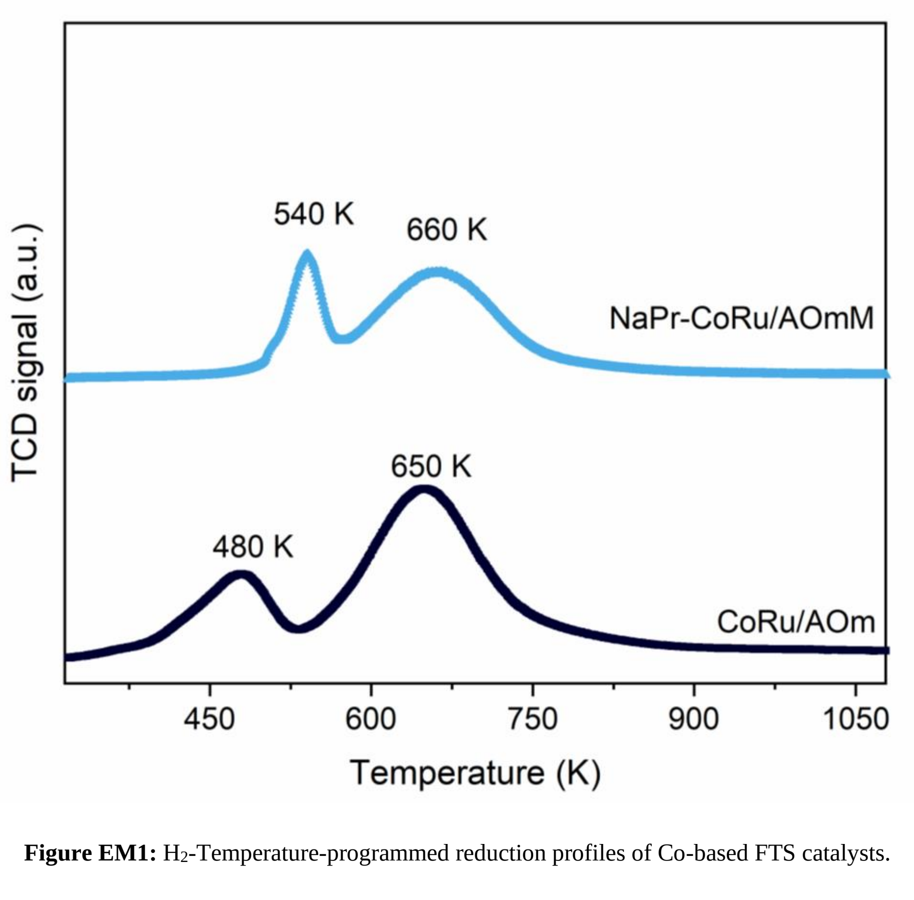

Figure EM1: H_2-Temperature-programmed reduction profiles of Co-based FTS catalysts.

1.2.4. H_2 chemisorption

Surface-exposed metal surface areas in supported solid FTS catalysts were determined using H_2 chemisorption uptakes at 373 K by plateau extrapolation to zero H_2 pressure.[1] Experiments were performed in an ASAP 2010C Micromeritics device. Prior to H_2 dosing, the oxidic sample (ca. 0.1 g of supported cobalt catalyst) was reduced *in situ* under flow of pure H_2 at 673 K for 5 hours (heating rate from RT of 2 K min^{-1}). After reduction, the sample was degassed at 1.3 Pa and the temperature lowered to 373 K to record the H_2 chemisorption isotherm. Co^0 surface areas were determined from the total amount of chemisorbed H_2, and the Co loading as determined by H_2-TPR. The contribution of Ru^0 to the overall metal content was considered insignificant. A surface H/Co=1 stoichiometry and a surface atomic density of 14.6 Co_{at} nm^{-2} were considered.

1.2.5. Chemical analysis by Energy-dispersive X-ray spectroscopy (EDS)

In order to quantify bulk promoter contents in promoted FTS catalyst samples, energy-dispersive X-ray spectroscopy (EDS) was performed on a Hitachi S-3500N scanning electron microscope. The powder sample was ground and then applied onto a pin-stub SEM mount coated with double-adhesive-face conductive carbon-tab. EDS spectra of areas of 1 mm^2 were scanned using an Oxford Pentafet 10 mm^2 detector to make sure a statistically relevant number of catalyst particles was jointly analyzed.

1.2.6. Tomographic Focused-Ion-Beam Scanning-Electron Microscopy (FIB-SEM)

Catalyst microparticles (80-100 μm) were embedded in a low-viscosity epoxy resin (Spurr) and the resin cured at 343 K for 12 h. The resin-embedded sample block was then trimmed and polished in an ultramicrotome (Reichert Ultracut) using first a glass knife (coarse trimming) followed by a fine trimming with a diamond knife (Diatome). Finally the trimmed specimen was mounted on a SEM stub with conductive colloidal graphite adhesive. The stub-mounted sample was sputter-coated with a ca. 20 nm carbon overlay using a LEICA EM MED020 sputter coater to achieve full conductive joints and minimize local charging artefacts during SEM imaging. Focused Ion Beam-Scanning Electron Microscopy (FIB-SEM) experiments were performed in a Zeiss Auriga Compact dual-beam microscope. First, a protective Pt layer was deposited on the region of interest (ROI) using the gas injection system. Then, the Ga^+ ion gun, operated at an intensity of 10 nA, was used to mill a front and two lateral trenches, delimiting the volume to be imaged, as well as a cross fiducial marker on the top surface

of the ROI to be used for automated image recognition and drift correction during the slice-and-image procedure. Serial sectioning combined with SEM imaging was carried out with specimen drift and beam shift corrections. Slices with nominal thickness of 60 nm were milled off using the Ga^+ FIB gun operated at an intensity of 3 nA. The corresponding SEM micrographs of the consecutively exposed cross-sections were recorded with a secondary electron detector while the electron gun was operated at a voltage of 2 kV.

The stack of raw micrographs was corrected for the foreshortening caused by the tilt angle between the specimen cross-section and the SEM detector (54° in the dual beam microscope employed in this study). Next, a stack de-flicking filter was applied to correct inter-micrograph gray-value gradients within the stack of images. After stack alignment using an iterative cross-correlation algorithm, the reconstructed tomograms were segmented using a watershed algorithm in Imagej,[2] followed by fine adjustment of the automatically recognized volumes via controlled erosion-dilation functions, to remove artefact material "islands", as well as manual threshold adjustment to correct for local gray-scale gradients created by either curtaining effects or shadowing phenomena. After tomogram binarization, a 3D local Euclidean distance transform[3] was propagated along the three-dimensional skeleton of the "solid" alumina phase to quantify the shortest distances from any point within the mesoporous alumina domains to the nearest boundary to the macropore network in Avizo (Thermo Fischer Scientific), where volumes were also surface-rendered for 3D visualization. There so-derived Euclidean mesopore transport distances were corrected for a pore tortuosity (τ) factor estimated as $\tau = \frac{\varepsilon}{1-(1-\varepsilon)^{2/3}}$,[4] where ε is the void fraction of the material's mesoporosity derived from the mesopore volume as $\varepsilon = \frac{1}{1+\frac{1}{\rho \cdot PV_{meso}}}$, where ρ is the materials skeletal density and PV_{meso} is the mesopore volume as determined by Hg intrusion porosimetry.

1.2.7. High-Angle Annular Dark-Field Scanning-Transmission Electron Microscopy (HAADF-STEM)

HAADF-STEM was applied to image the nanostructure of solid FTS catalysts. Before microscopy observation, samples were embedded in a low viscosity resin (Spurr, hard composition) and hardened at 343 K. Then nanometer thin slices (nominal thickness of 150 nm) were obtained with a Diatome diamond knife mounted on a Reichert Ultracut ultramicrotome and collected on a copper TEM grid (300 mesh) covered with a lacey carbon film. High-angle annular dark-field (HAADF) STEM micrographs and Energy Dispersive X-ray Spectroscopy (EDS) elemental maps were acquired using a beam spherical aberration-corrected (C_s) Hitachi HD-2700

dedicated Scanning Transmission Electron Microscope (STEM) equipped with a cold field-emission gun and two EDAX Octane T Ultra W EDS detectors (Ametek) and operated at 200 kV, and a Jeol JEM 2100F Scanning Transmission Electron Microscope (STEM) equipped with a X-Mac EDS detector (Oxford instruments) and operated at 200 kV.

1.3. Inductively-coupled-plasma mass-spectrometry and optical-emission spectroscopy.

Elemental (Co, Na, Pr) contents in selected reaction liquors were determined by inductively-coupled-plasma mass-spectrometry (ICP-MS). To digest the organic material, 10 mg of sample were mixed with 8 mL concentrated HNO_3 and heated in a microwave oven (CEM, Mars 6) up to 200 °C (1500 W, temperature ramp of 30 minutes from RT). Afterwards the temperature was held for 15 minutes. After cooling down, the sample was filled up to 100 mL with H_2O. After acid digestion, the sample was injected into the ICP-MS (ICPMS-2030, Shimadzu) and measured at least two times. Before each measurement, commercially available calibration standards (Carl Roth) for the target element, in the expected concentration range, where injected similarly. The metal/lanthanide concentration in the organic reaction liquors was determined by comparing the average of all measurements with the pre-measured calibration curve. The concentration in the liquor samples was referenced to the background concentration determined in a preparation blank solution that has been taken through an identical sample preparation procedure. The Na concentration in the preparation blank was already too high for a reliable determination by ICP-MS. Hence, Na analysis was also performed by inductively-coupled-plasma optical-emission-spectroscopy (ICP-OES) in a iCAP PRO spectrometer (Thermo Scientific) calibrated with certified Na standards. The absence of Na leaching into solution was inferred from experimental Na concentrations in the liquid samples statistically identical to, or lower than, those in the preparation blank (0.2 ppm).

1.4. Nuclear Magnetic Resonance

The structure of molecular olefin reductive hydroformylation catalysts was assessed by $^{1}H[^{31}P]$ and $^{31}P[^{1}H]$ nuclear magnetic resonance (NMR) spectroscopy. For this, between 50 mg and 300 mg of the solution after the specific reaction procedure were transferred under inert atmosphere into a NMR young-tube and diluted with 200 mg to 500 mg of the selected deuterated solvent. Spectra were acquired on a 500 MHz NMR spectrometer (Bruker) equipped with an auto sampler at 293 K. $^{1}H[^{31}P]$-NMR measurements were collected with at least 128

scans each, in order to identify hydride signals of catalyst species in low concentrations.

1.5. Catalyst testing in the Fischer-Tropsch synthesis (FTS)

The catalytic performance of supported cobalt-based catalysts was assessed by means of gas-phase Fischer-Tropsch synthesis tests. The FTS catalytic tests were performed at 20 bar and 473 K in a fixed-bed reactor setup. The setup is equipped with mass-flow controllers (Bronkhorst, MFCs) to feed H_2 (Air Liquide, 99.999%, CAS: 1333-74-0) and pre-mixed syngas (30 vol.% CO, 60 vol.% H_2, 10 vol% Ar as internal standard, Air Liquide, 99.99%), two-finned copper elements with embedded 180 W heating cartridges to heat the stainless steel (316L grade) fixed-bed micro-reactor (inner diameter, *i.d.* = 12 mm), two thermocouples (K-type, 0.5 mm) placed at the start and at the end of the catalyst bed as part of two PID feedback loops to control the temperature inside the reactor, and an online gas chromatograph (modified Agilent 7890B) for product analysis.

Upstream of the syngas MFC, the syngas stream was first passed over an activated carbon bed (Norit) at 30 bar and room temperature to remove metal-carbonyl impurities that might be entrained from the pressurized gas cylinder, and then through a 316L grade stainless steel (length, *l* = 300 mm, *i.d.* = 3 mm inner diameter) capillary loop maintained at 473 K to pre-heat the syngas. The free volume in the reactor was reduced by inserting two 316L grade stainless-steel spacers, one (*l* = 62 mm) upstream of the catalyst bed, and another (*l* = 50 mm) downstream of the bed. The spacers were separated from the catalyst bed using two quartz wool plugs (*l* = 5 mm). Below the first quartz wool plug, a 3.4 cm^3 layer of SiC granules (Alfa Aesar, 46 grit, CAS: 409-21-2) was placed to additionally pre-heat syngas and help establish plug-flow before entering the catalyst bed. The catalyst bed consisted of an amount of catalyst equivalent to 180-300 mg of Co^0 as determined by H_2-TPR. This amount of supported catalyst (Ø = 0.08-0.10 mm) was diluted with 6.2 cm^3 of SiC granules (Alfa Aesar, 46 grit, 409-21-2) to improve heat transfer in the catalyst bed.

Before the FTS tests, the metal species in the catalyst were reduced in H_2 flow (200 cm^3 STP min^{-1}) at 673 K (2 K min^{-1} to 423 K, followed by 0.83 K min^{-1} to 673 K) for 5 h at atmospheric pressure. This reduction temperature was selected on the basis of the H_2-temperature programmed reduction profiles registered for the herein reported FTS catalysts (Figure EM1). Following catalyst reduction activation, the reactor was cooled to 423 K in H_2 flow, followed by switching the flow to the synthetic syngas mixture (150 cm^3 STP min^{-1}; weight

hourly space velocity, *WHSV* = 3.6–11.0 h^{-1}). The system was then pressurized to 20 bar using a membrane dome regulator (GO regulator), and the reactor temperature was increased to 473 K (0.15 K min^{-1}). The start of the temperature ramp from 423 K to 473 K in syngas was marked as time-on-stream = 0 h. Both reactor design and startup protocols have been optimized to ensure an isothermal catalyst bed operation. After at least 24 h time-on-stream, the syngas flow was adjusted to determine catalyst performance at a 20±3% CO conversion level.

Downstream of the reactor, two consecutive cold traps were set at temperatures of 423 K and 373 K at the reaction pressure to collect heavy hydrocarbon products and water. All other downstream tubing was heated to 443 K to prevent the condensation of reaction products. All gaseous components of this gas stream leaving the traps were depressurized after passing the dome pressure regulator and were analyzed by online gas chromatography. The gas chromatograph is equipped with two sampling loops, one of which feeds into a capillary column (Restek RTX-1, 60 m) equipped with an FID detector, while the other feeds into two consecutive packed-bed columns (HS-Q 80/120, 1x m + 1x 3 m) equipped with a TCD for the analysis of H_2, CO_2, and C_2-C_3 hydrocarbons. Along this analysis channel, a molecular sieve column is used for the separation of Ar, CH_4, and CO, which are detected using an additional TCD. Figures EM2 and EM3 show representative product chromatograms obtained online for the gas product stream collected while testing a dually promoted NaPr-CoRu/AOmM meso-macroporous CoRu/γ-Al_2O_3 catalyst. CO, CH_4, and CO_2 were quantified using TCD response factors relative to Ar, whereas for usual tests C_{2+} hydrocarbons and alcohols were quantified in the FID relative to CH_4. CO conversion (X_{CO}) and the metal-specific reaction rate (cobalt-time-yield (CTY)) were used to express catalytic activity (Equations 1 and 2). Selectivity to C_{5+} hydrocarbon products was determined by deducing the selectivity to C_{4-} carbon-containing products, including CO_2, from a closed carbon balance (Equations 3 and 4). Liquid and solid hydrocarbons collected from the trap kept at 373 K and 20 bar were separated by decanting, the individual liquid phases were further purified by centrifugation (9000 rpm, 15 min) to collect waxes and obtain clear polar and non-polar phases which were weighed separately. Solid wax hydrocarbons collected from the trap kept at 423 K and 20 bar were blended together with those wax fractions collected by centrifugation of the liquid products, weighed, and dissolved in CS_2 (≥99%, Sigma-Aldrich, CAS: 75-15-0). Acetone (HPLC grade, ≥99.9%, Sigma-Aldrich, CAS: 67-64-1) was added in the case of aqueous phase samples and 2-methyl heptane (>98.0%, Sigma-Aldrich, CAS: 592-27-8) was added in the case of non-polar oil and wax solution samples as gas chromatography standards, respectively. Offline gas chromatography of the organic hydrocarbon samples was performed on a second Agilent 7890B gas chromatograph equipped with a capillary column (Restek RTX-1, 60 m) which elutes into an FID. Offline gas chromatography of

aqueous phase products was performed on a third Agilent 7890B gas chromatograph equipped with a high-polarity polyethylene glycol (DB-Waxetr, iD=0.25 mm, film thickness=0.25 µm, 30 m) which elutes into an FID. Carbon balances closed at 96.5±2.0%. For catalyst reuse tests, following a first tandem FTS/RHF reaction run (24 h), the reaction liquors were removed from the reactor through an immersion capillary equipped with a (Type 113P, 5-8 µm, Carl Roth GmbH) filter to retain solid particles, also employing a riser tube to enable gas flow. Liquor removal was performed at the reaction temperature (hot filtration) to provide a fair assessment of catalyst stability, avoiding artifacts associated to possible metal re-deposition phenomena upon cooling the reaction slurry. The solid catalyst was recovered and reused for a second FTS/RHF test after replenishing the reactor inlet with fresh solvent, RHF catalyst, and syngas. All tasks were performed under air exclusion in an Ar-filled glovebox to prevent metal re-oxidation. No catalyst rejuvenation/regeneration treatment was applied between the consecutive runs. Following the second run, the exact amount of FTS catalyst was determined in order to correct activity values for any loss of FTS catalyst during filtration and re-slurrying between runs. To this end, the solid was isolated by ultracentrifugation and the catalyst mass determined as the solid residue by TGA.

$$CO\ conversion\ (X_{CO}) = \left(1 - \frac{\dot{n}_{CO,out}}{\dot{n}_{CO,in}}\right) \cdot 100 \quad \text{(Equation 1)}$$

$$Cobalt\ time\ yield\ (CTY) = \dot{n}_{CO,in} \cdot X_{CO} \cdot m_{Co}{}^{-1} \quad \text{(Equation 2)}$$

$$S_{C1-C4} = \sum_{i=1}^{4} \dot{n}_{CO,out,i} \cdot n_i \div (\dot{n}_{CO,in} - \dot{n}_{CO,out}) \cdot 100 \quad \text{(Equation 3)}$$

$$S_{C5+} = 1 - S_{C1-C4} \quad \text{(Equation 4)}$$

wherein:

$$\dot{n}_{CO,in} = molar\ flow\ of\ inlet\ CO$$

$$\dot{n}_{CO,out} = molar\ flow\ of\ CO\ detected\ by\ GC$$

$$m_{Co} = cobalt\ mass$$

$$n = carbon\ number\ of\ a\ detected\ hydrocarbon$$

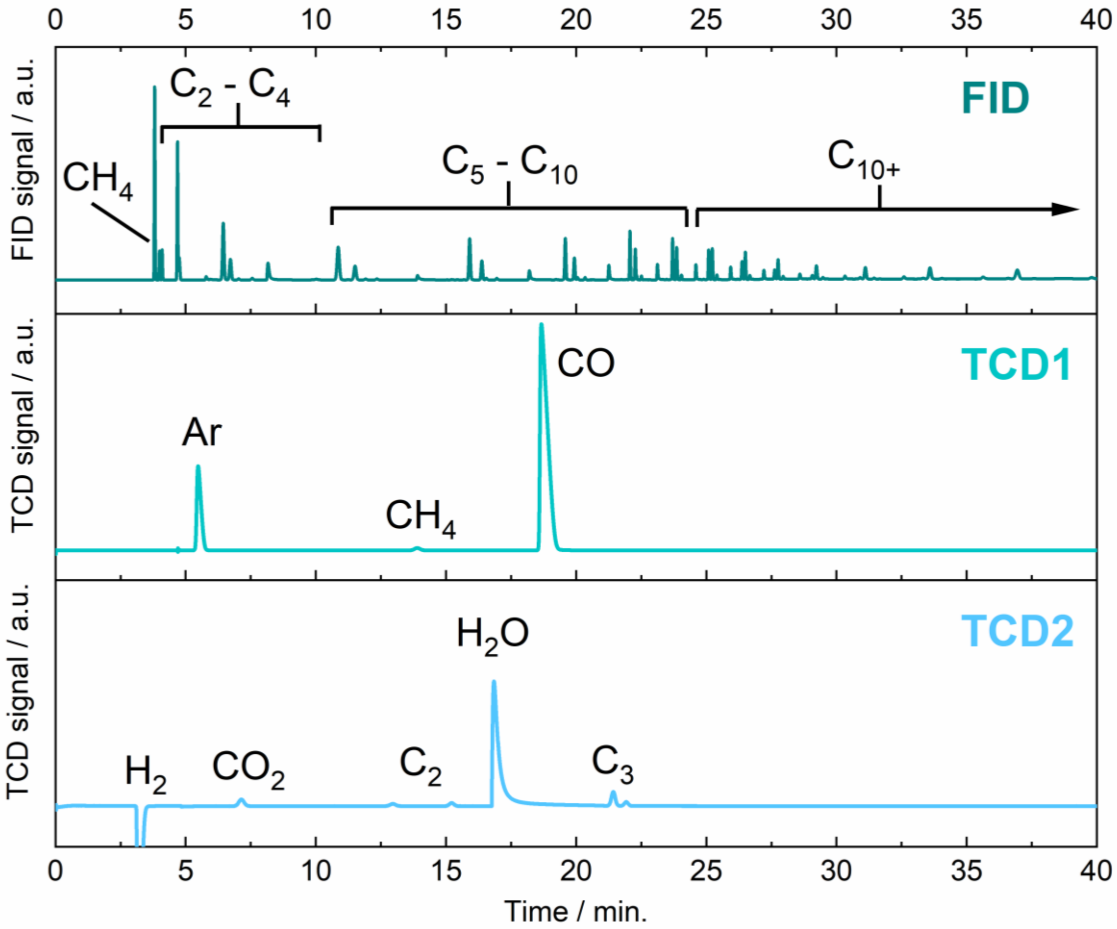

Figure EM2: Representative online product chromatograms for a fixed-bed FTS catalytic test with NaPr-CoRu/AOmM. a) Hydrocarbon products in the range of C_1-C_{16} eluting from a Restek RTX-1 capillary column can be detected in the flame-ionization detector (FID) of the Agilent GC7890B. Permanent gases eluting from a 13X molecular sieve packed column are detected on a first TCD (TCD1), whereas light hydrocarbon products (C_2-C_3), as well as CO_2 and water eluting from two consecutive HS-Q 80/120 packed columns are detected on a second TCD (TCD2).

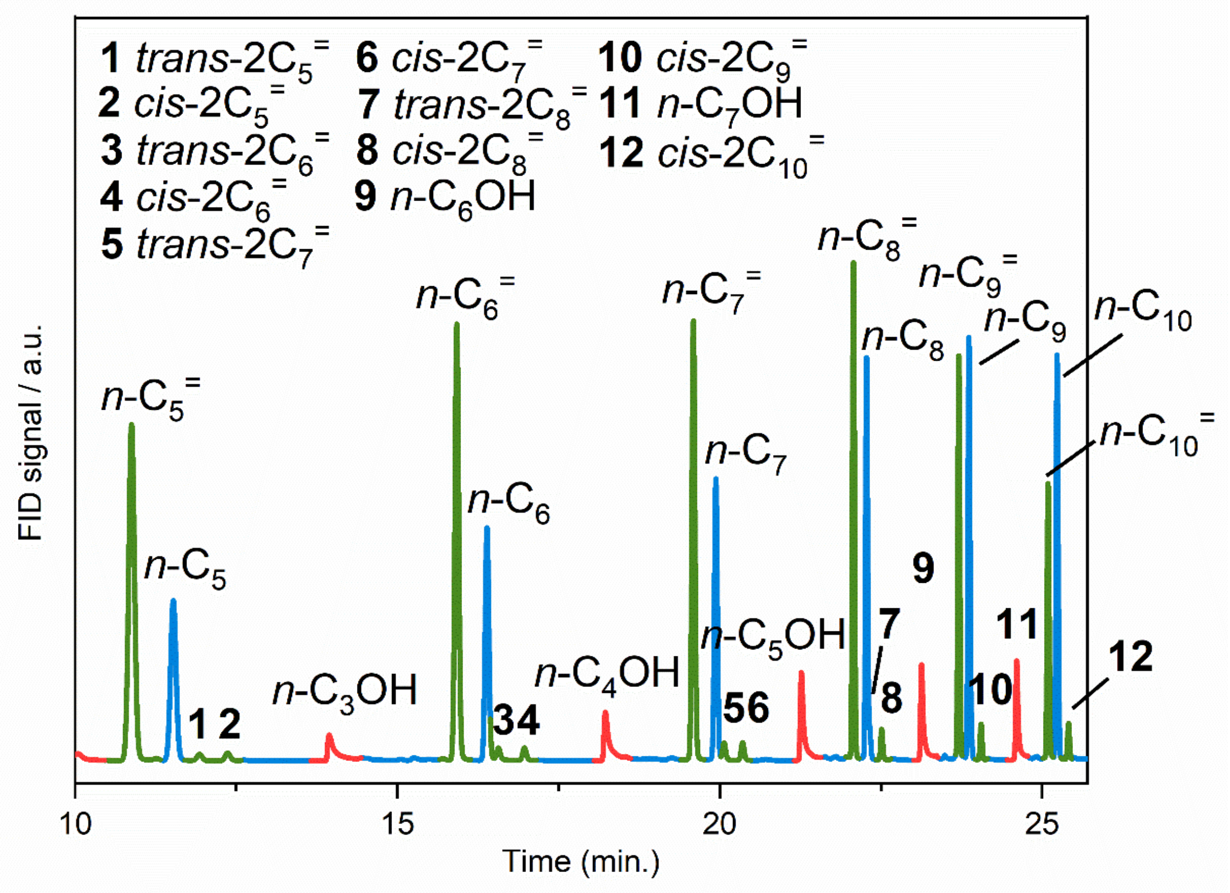

Figure EM3: Detail view of C_5-C_{10} hydrocarbon and oxygenate products from a fixed-bed FTS catalytic test with NaPr-CoRu/AOmM as detected online in the FID of the gas chromatograph downstream of the FTS testing setup, highlighting the predominance of α-olefin products in the C_{5-10} range. Trans-$C_9^=$ and trans-$C_{10}^=$ are overlapped by *n*-C_9 and *n*-C_{10}, respectively. Color code: Green (*n*-olefin products, *n*-$C_n^=$ for 1-olefins and $2C_n^=$ for 2-olefin isomers), blue (*n*-paraffin products, *n*-C_n), red (*n*-alcohol products, *n*-C_nOH).

2.1 General experimental procedure for the reductive hydroformylation reaction of *1*-octene

The performance of cobalt-based RHF catalysts was evaluated in a wide range of operational settings in hydroformylation tests using *1*-octene as model compound for mid-chain linear FTS 1-olefin products. This set of experiments was performed as follows. Under inert atmosphere the dicobalt octacarbonyl catalyst precursor ($Co_2(CO)_8$, Acros organics, 95 % purity, CAS 10210-68-1) and the corresponding phosphine ligand, i.e. tricyclohexyl phosphine (PCy_3), Acros organics, 97 %, 2622-14-2; or tri-*n*-butyl phosphine (P*n*-Bu_3), ABCR GmbH, 99 %, CAS 998-40-3; or triphenyl phosphine (PPh_3), Alfa Aesar, 99 %, CAS 603-35-0; or polymer-bound triphenyl phosphine (phenylphosphinated copolymer of styrene and divinylbenzene (PPh_3 loading of 3.2 mmol/g, abbreviated as Poly-PPh_3), Sigma Aldrich, CAS 39319-11-4, were weighed into a borosilicate glass inlet of a 10 mL steel autoclave, equipped with a magnetic stirring bar under inert conditions (mBraun UNIlab ECO Glovebox with argon atmosphere, <0.1 ppm H_2O, <4 ppm O_2) . Afterwards, *1*-octene (TCI chemicals, 97 %, CAS 111-66-0), 0.1 g tetradecane (ABCR GmbH, 99 %, CAS 629-59-4) as internal standard for gas chromatography and 2.75 mL of the designated solvent, i.e. (typically 2-methyl pentane (Sigma Aldrich, >99 %, CAS 107-83-5), alternatively 2-methyl butane (Supelco, 99.8 %, CAS 78-78-4) were added. The glass inlet was inserted into the reactor, the reactor sealed and flushed with argon. After flushing at least three times with hydrogen (Air Liquid, 99.999 %, CAS 1333-74-0), the reactor was pressurized to the designated partial pressure of hydrogen (P_{H2}) in the desired syngas composition. Afterwards carbon monoxide (Air Liquid, 99.997 %, CAS 630-08-0) was dosed to reach the designated partial pressure of CO (P_{CO}) in the desired syngas composition. After pressurizing, the autoclave was heated to the desired reaction temperature (typically 468 K) in a (preheated) aluminum block fitted on a magnetic stirrer. Reaction was let to proceed in batch mode under vigorous magnetic stirring (700 rpm). Once the reaction time had elapsed, the autoclave was allowed to cool to RT and the pressure was released. Post reaction analysis of the liquid phase was performed *via* gas chromatography in a Shimadzu Nexis GC-2030 chromatograph equipped with a Restek RTX-1 column (30 m, 0.25 mm ID, 0.5 μm film thickness) and a Flame-Ionization-Detector (FID). For analysis, 0.2 mL of the reaction solution was weighed into a GC vial and diluted with *iso*-propanol. Product quantification was performed using the tetradecane internal standard as reference and FID response factors determined from the corresponding pure compounds.

2.2 General experimental procedure for slurry-phase tandem Fischer-Tropsch synthesis/reductive hydroformylation (FTS/RHF) experiments

Tandem Fischer-Tropsch synthesis/reductive hydroformylation catalytic experiments were performed in slurry phase. Experimentally, the solid Fischer-Tropsch catalyst (previously activated by reduction in H_2 flow at 673 K for 5 h and stored in a glove box under exclusion of air) was weighed into the borosilicate glass inlet of a 10 mL steel autoclave, equipped with a magnetic stirring bar. To this, the corresponding amounts of dicobalt octacarbonyl ($Co_2(CO)_8$), organo-phosphine ligand and solvent were added. 2-methyl heptane (~50 mg) was added as internal standard for gas chromatography quantification of liquid products. All substances were weighed and added under protective inert atmosphere (mBraun UNIlab ECO Glovebox with argon atmosphere, <0.1 ppm H_2O, <4 ppm O_2). Afterwards the autoclave was sealed and pressurized to the initial total pressure of 120 bar at RT, with a synthetic syngas mixture containing 10 vol% Ar N50, 30 vol% CO N18, rest H_2 N30 (Air Liquide), where Ar was added as an internal standard for gas chromatography quantification of headspace gases following termination of the reaction test. In order to assess the influence of a pre-forming step for the molecular RHF catalyst, in a set of experiments, the autoclave containing only the dicobalt octacarbonyl and ligand precursors in the solvent phase was sealed, pressurized to 120 bar with the synthetic syngas mixture and heated to the reaction temperature for 1 h. Then, the autoclave was allowed to cool down to RT, the headspace gas vented to reach a total pressure below 10 bar, opened under exclusion of air and transferred back into an Ar-filled glove box, where the pre-activated solid FTS catalyst and 2-methyl heptane as internal GC standard were added into the glass inlet of the reactor, any loses of solvent due to the flushing of the reactor in the glove-box antechamber replenished, and the reactor sealed and re-pressurized again to the initial total pressure of 120 bar with the synthetic syngas mixture. In either case, following syngas dosage, the autoclave was heated to the desired reaction temperature (typically 473 K) in a (preheated) aluminum block fitted on a magnetic stirrer. Reaction was let to proceed in batch mode under vigorous magnetic stirring (700 rpm). Once the reaction time had elapsed, the autoclave was allowed to cool to RT.

The headspace gas was analyzed by releasing a portion of the gas phase after the reaction to flush and fill two sampling loops of an Agilent 7890B gas chromatograph (GC) connected in series. One of those sampling loops is then injected into a capillary column (Restek RTX-1, 60 m) that elutes into a Flame-Ionization detector (FID), while the other sampling loop is injected into two consecutive packed-bed columns (HS-Q 80/120, 1x m + 1x 3 m), that elute into a Thermal-Conductivity detector (TCD) for H_2, CO_2, and C_2-C_3 hydrocarbons quantification. Further down that analysis channel, a molecular sieve column (13X) separates Ar, CH_4, and CO

permanent gases, by means of a second TCD. After sample injection, the GC oven temperature was kept at 308 K for 10 min, after which the oven was heated to 483 K (heating ramp 283 K min^{-1}). Then, the temperature was kept at 483 K for 45 min. CO, CH_4, and CO_2 were quantified using TCD response factors relative to Ar, whereas C_{2+} hydrocarbons and alcohols were quantified in the FID relative to CH_4.

Condensed phases in the reactor after the reaction test were collected and then centrifuged in an ultracentrifuge at 13.000 rpm, after which the liquid and solid phases were separated and recovered separately. The liquid sample was injected into an Agilent 7890B GC equipped with a capillary column (Restek RTX-1, 60 m) which elutes into an FID. Following injection, the GC oven temperature was heated from RT to 493 K (heating rate 278 K min^{-1}), after which the temperature was kept at 493 K for 50 min. Hydrocarbons and alcohols were quantified based on their FID signal relative to a known amount of iso-octane as internal standard.

For selected tests, the liquid product fraction was additionally analyzed by two-dimensional gas chromatography in a 2D GCxGC LECO Pegasus 4D chromatograph equipped with a flame ionization detector (FID) and a time-of-flight-mass spectrometer (TOF-MS) using an analysis protocol previously optimized for complex mixtures of hydrocarbons and higher oxygenates.[5] The system was operated in reverse phase configuration, where the first dimension is a polar column (Stabilwax) and the second dimension is a non-polar (RTX-5), see Table EM1 for detailed analysis conditions and column specifications. Modulation of the effluent from the primary column was achieved by cooling a dry N_2 gas stream with liquid nitrogen. Data analysis was performed using the TOF Chrom software package.

Table EM1: 2D GCxGC detailed analysis conditions and column specifications.

2D GCxGC	
**Injection method**	Liquid injection (0.2 µL)
**Mode**	Split
**Injector temperature**	250 ˚C
**Split ratio**	100
**Column**	
**1st dimension**	*Stabilwax*, 30 m, 250 µm, 0.1 µm.
**2nd dimension**	*RTX-5*, 1.39m, 180 µm, 0.2 µm.
**Temperature program**	60 ˚C hold 1 min<br>60 ˚C to 240 ˚C at 5 ˚C/min hold time 5 min
2D GC-FID	
**Total flow rate**	2.1 ml/min
**Detector temperature**	280 ˚C
**Modulator**	
**Temperature off set**	+35 ˚C
**Modulation period**	8 seconds
**Hot pulse**	2 seconds
**Cold pulse**	2 seconds
2D GC-MS	
**Total flow rate**	1.1 ml/min
**Ionization source temperature**	225 ˚C
**Modulator**	
**Temperature off set**	+35 ˚C
**Modulation period**	10 seconds
**Hot pulse**	2.5 seconds
**Cold pulse**	2.5 seconds

The amount of waxy hydrocarbons in the solid pellet recovered after centrifugation was quantified by thermogravimetric analysis (TGA) in air using corundum crucibles in a Netzsch Jupiter STA 449 C thermobalance and exposing the sample to a heating ramp of 10 K min^{-1} from 298 K to 1023 K. The fraction of the total amount of combusted matter associated to waxy hydrocarbon products was estimated by subtracting contributions from (i) the release of CO ligands from $Co_2(CO)_8$ on the basis of the cobalt carbonyl stoichiometry, the Co/Al stoichiometry in the Fischer-Tropsch catalyst and the total cobalt content in the inorganic residue left behind in the crucible after the TGA experiment as determined by energy-dispersive X-ray analysis (EDS) in a Hitachi S-3500N scanning electron microscope equipped with an EDS detector (Oxford Instruments); and (ii) the combustion of organic ligands from the hydroformylation catalyst as determined based on the total phosphorous content in the ligand and the ligand-to-cobalt stoichiometry in the molecular hydroformylation catalyst. Wax contents determined this way were <2 C% in all cases.

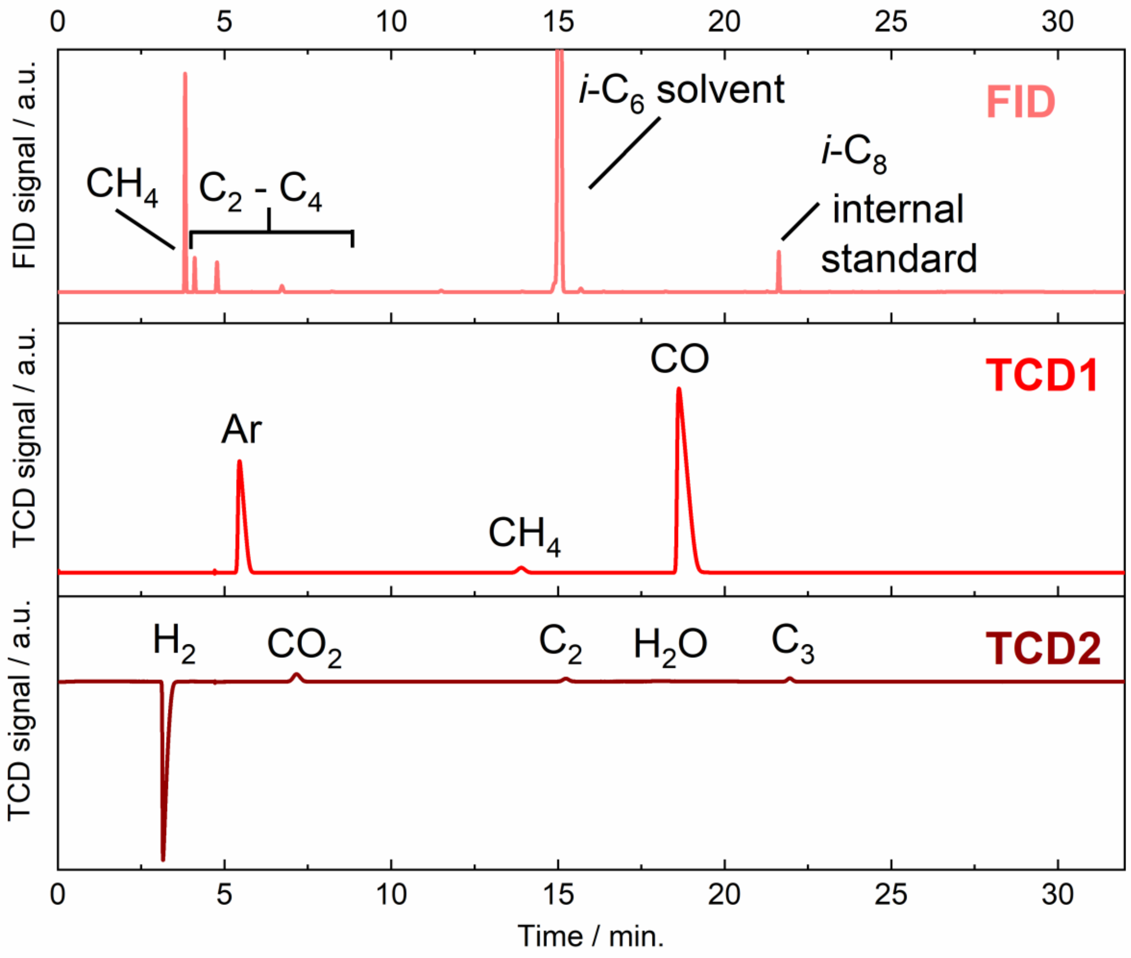

Figure EM4: Representative gas-phase (headspace) product chromatograms for a tandem FTS/RHF catalytic test in slurry phase. a) Hydrocarbon products in the range of C_1-C_5 eluting from a Restek RTX-1 capillary column can be detected in the flame-ionization detector (FID) of the Agilent GC7890B. Permanent gases eluting from a 13X molecular sieve packed column are detected on a first TCD, whereas light hydrocarbon products (C_2-C_3), as well as CO_2 and water eluting from two consecutive HS-Q 80/120 packed columns are detected on a second TCD.

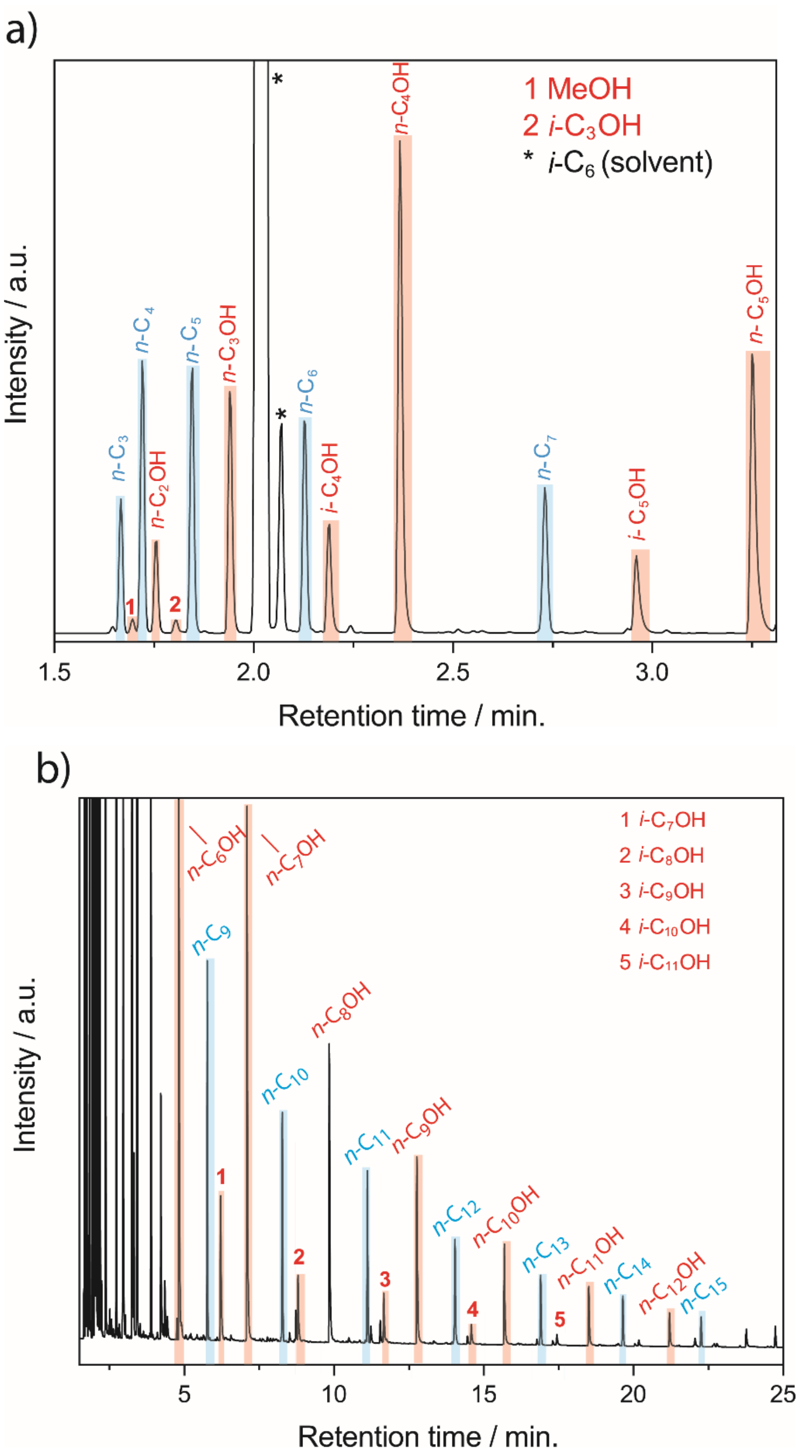

Figure EM5: Representative liquid-phase product chromatograms for a tandem slurry-phase FTS/RHF catalytic test. a) Detail view of hydrocarbon and alcohol products labeling *n*-alkanes in the range of C_3-C_7 and alcohols in the range of C_1-C_5. b) Overview of hydrocarbon and alcohol products labeling *n*-alkanes in the range of C_9-C_{14} and alcohols in the range of C_{6-12}.

3. Supporting Figures

a)

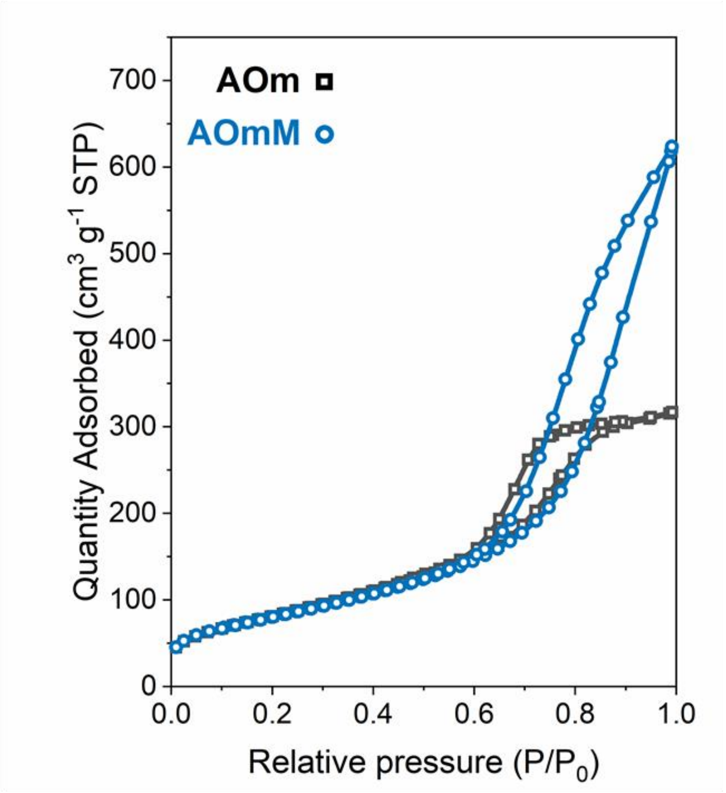

b)

γ-Al_2O_3 support	S $_{BET}$[a] [m^2 g^{-1}]	mesoPV[b] [cm^3 g^{-1}]	macroPV[c] [cm^3 g^{-1}]	meso*PD*[d] [nm]	macro*PD*[d] [nm]
AOm	300	0.50	0.11	8.2	-
AOmM	293	0.91	1.12	7.5	$2.4 \cdot 10^3$

[a] BET specific surface area determined by N_2-physisorption. [b] Mesopore volume as determined by the BJH formalism from the corresponding N_2 adsorption isotherms. [c] Macropore volume as determined by integration of the cumulative intrusion volume for pore diameters in the range of 50-$5.0 \cdot 10^3$ nm from the corresponding Hg intrusion results. [d] Major pore diameter modes as determined by the peaking position of maxima in the differential Hg intrusion pore size distribution.

Figure S1: Textural properties of γ-Al_2O_3 support materials applied in the synthesis of solid Fischer-Tropsch synthesis catalysts. a) N_2-physisorption isotherms. b) summary of textural properties.

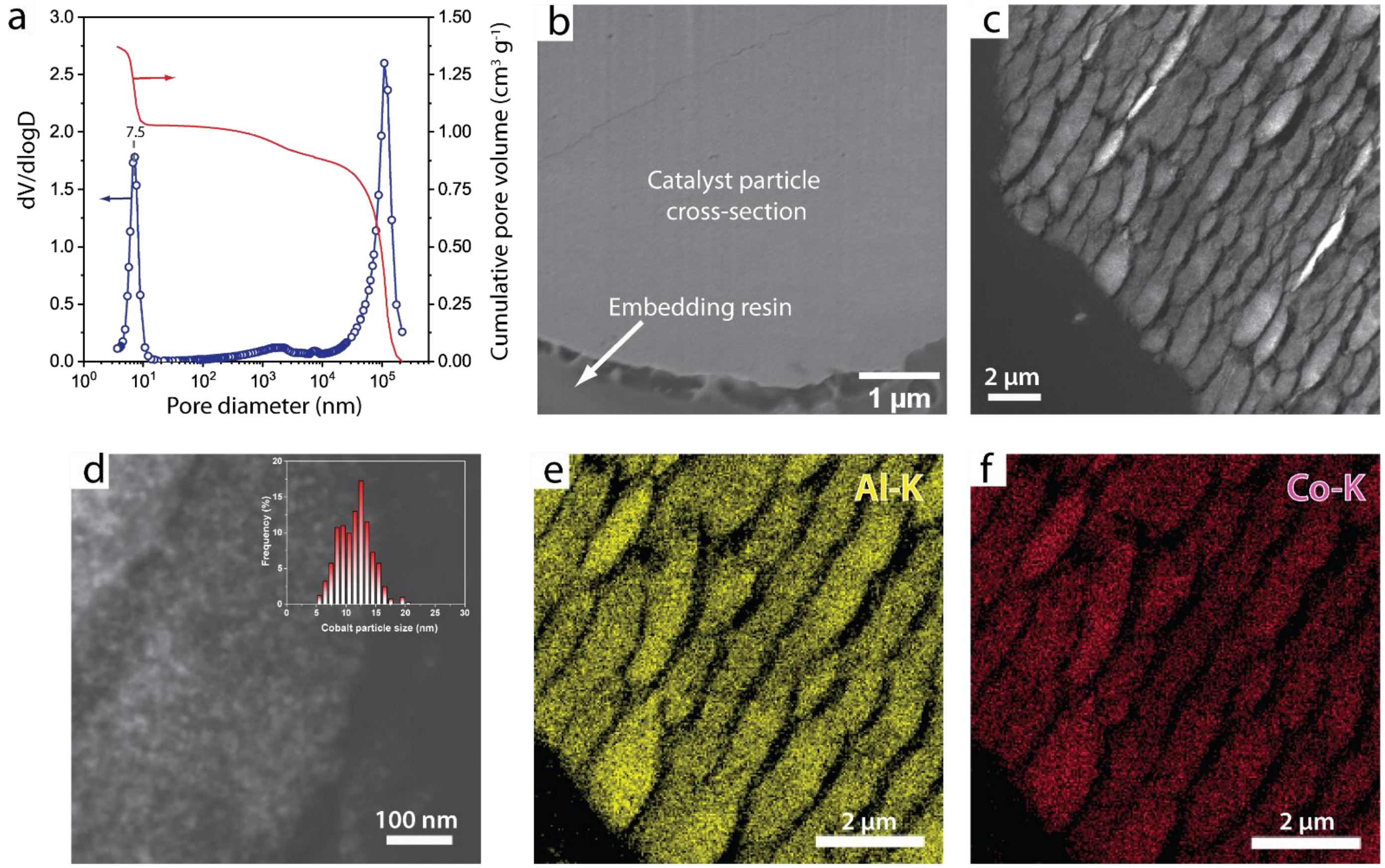

Figure S2: Structural characterization of the unpromoted cobalt-based Fischer-Tropsch catalyst supported on a strictly mesoporous-γ-Al_2O_3 carrier (CoRu/AOm). (a) Differential (blue scatter trace) and cumulative (red line) pore size distributions as determined by Hg intrusion porosimetry showing the absence of any remarkable microporosity in the pore diameter size range from 50-10^4 nm. The contribution detected at ca. 10^5 nm corresponds to voids between the catalyst microparticles; (b) Cross-sectional SEM micrograph after Focused-Ion-Beam (FIB) milling of the resin-embedded catalyst showing the absence of intra-particle macropore openings; (c,d) Representative low-magnification C_S-HAADF-STEM micrograph for an ultramicrotomed catalyst cross section after reduction activation and surface passivation. (d) High-magnification C_S-HAADF-STEM micrograph showing cobalt nanoparticles (brightest speckles with higher Z-contrast) confined to the network of γ-Al_2O_3 nanocrystallites. The inset shows the corresponding Co nanoparticle size histogram. (e,f) EDS microanalysis maps recorded at the Al- and Co-K emission lines, respectively, showing a spatially uniform distribution of the metal across the alumina support. Parallel stretch marks observed across the catalyst particle in panels c, e and f are created during the ultramicrotome sectioning of the resin-embedded specimen and do not correspond to intrinsic porosity of the catalyst.

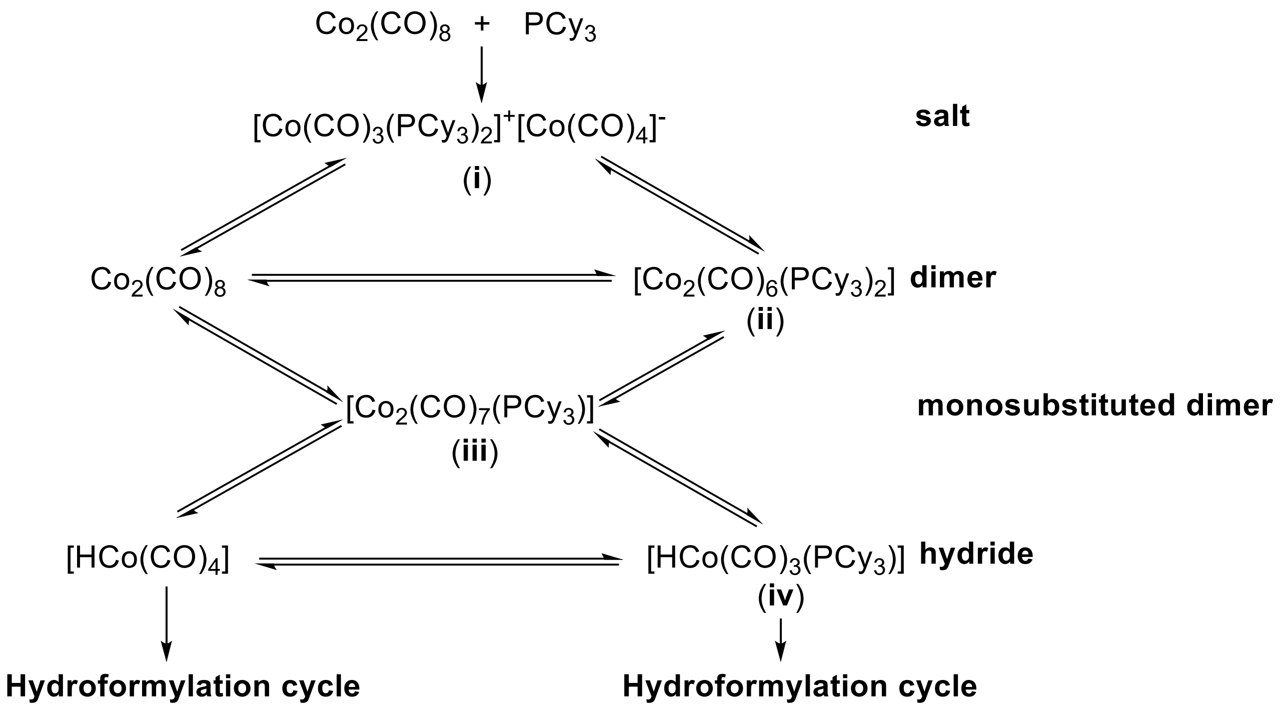

Figure S3: Pre-catalyst species equilibria for the $HCo(CO)_3(PCy_3)$ reductive hydroformylation catalyst. Adapted from[6].

Figure S3 shows a schematic representation of the proposed equilibria established between different species in a process of reductive hydroformylation (RHF) catalyzed by a trialkylphosphine-modified cobalt carbonyl RHF catalyst. It is generally acknowledged, that dicobalt octacarbonyl ($Co_2(CO)_8$) reacts rapidly with the phosphine ligand PCy_3 to generate a disubstituted salt, namely $[Co(CO)_3(PCy_3)_2]^+[Co(CO)_4]^-$ (Figure S3, **(i)**). At typical RHF operation temperatures, the latter salt converts rapidly into a bis(phosphine) dimer $[Co_2(CO)_6(PCy_3)_2]$ **(ii)** by further displacement of CO, as reported by *Brown* and co-workers.**[6b]** Since the formation of the coordinatively unsaturated hydride $[HCo(CO)_3(PCy_3)]$ **(iv)** is considered to be the rate-limiting step, only the pre-catalyst equilibria can be studied by means of NMR spectroscopy, as described by *Dwyer* and co-workers.[6a]

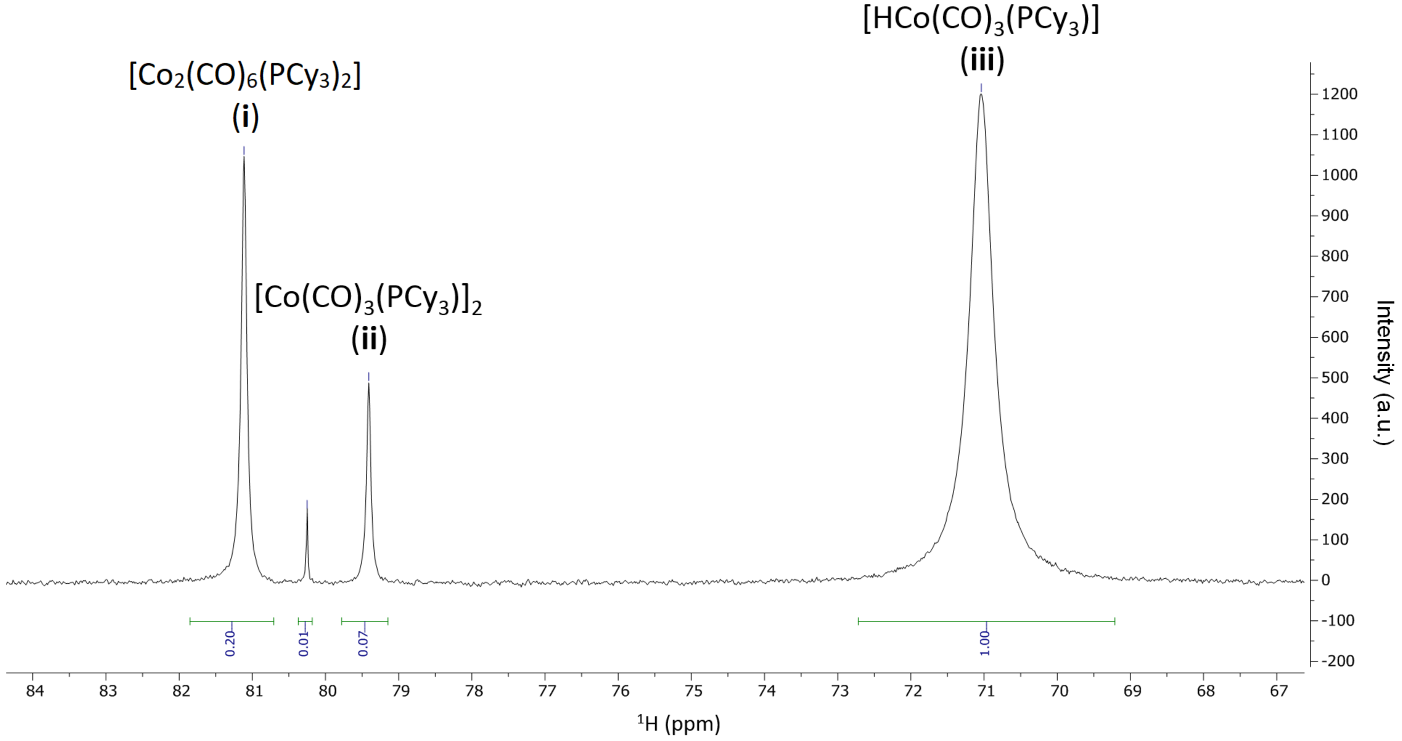

Figure S4: Analysis of molecular RHF catalyst species in the absence of solid FTS catalyst. $^{31}P[^{1}H]$-NMR (202 MHz, $CDCl_3$, RT) spectrum after the reaction of $Co_2(CO)_8$ and PCy_3 (M:L = 1:1) at 468 K for 1 h and 120 bar pressure (measured at RT) of syngas (H_2:CO = 2) including the assignment of the peaks based on chemical shifts as found in literature and chemical shifts of synthesized and isolated intermediates.

Figure S4 shows the $^{31}P[^{1}H]$-NMR spectrum recorded for a RHF reaction liquor in the absence of a Fischer-Tropsch catalyst. A solution of Co_2CO_8 and PCy_3 (Co(M):P(L) atomic ratio of 1:1) in *n*-hexane was exposed to RHF conditions for one hour (see caption to Figure S4 for experimental details). Afterwards NMR samples were taken under inert conditions. In good agreement with literature, the ^{31}P-spectrum shows a broad singlet at δ = 71.0 ppm which can be assigned to the hydride species $[HCo(CO)_3(PCy_3)]$ (Figure S4, (**iii**)). Besides, two species in lower concentrations with chemical shifts of δ = 79.4 ppm and δ = 81.1 have been identified as the intermediate dicarbonyl complexes $[Co_2(CO)_7(PCy_3)]$ (Figure S4, (**i**)) and $[Co_2(CO)_6(PCy_3)_2]$ (Figure S4, (**ii**)), respectively. Both complexes have been synthesized and isolated to confirm signal assignments, following procedures described by *Wood* and *Garrou*.[7] A singlet in very low concentrations at δ = 80.25 ppm remains unassigned.

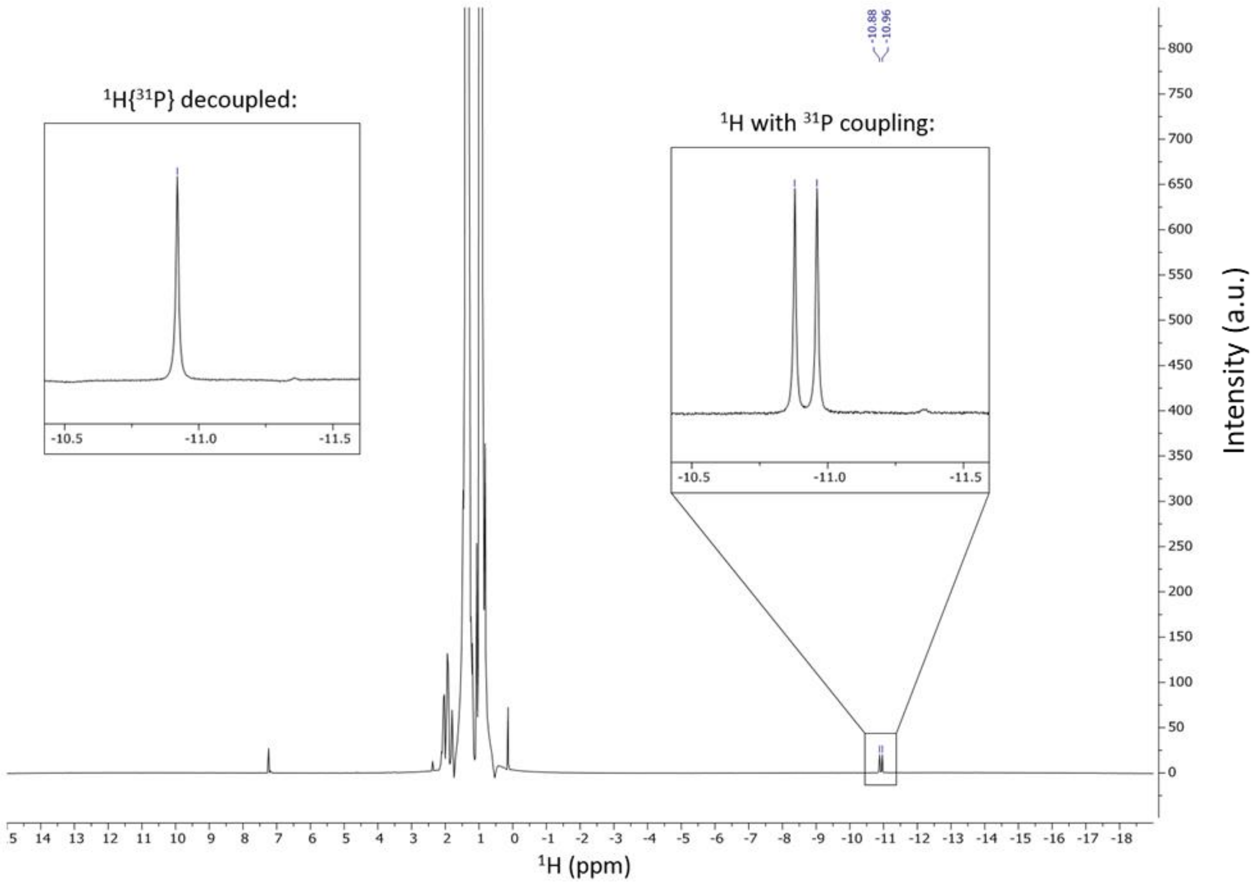

Figure S5: Analysis of molecular RHF catalyst species in the absence of solid FTS catalyst. ^{1}H-NMR (500 MHz, $CDCl_3$, RT) and ^{1}H[^{31}P] (500 MHz, $CDCl_3$, RT) spectrum after the reaction of $Co_2(CO)_8$ and PCy_3 (M:L = 1:1) in hexane at 468 K for 1 h and 120 bar (measured at RT) pressure of syngas (H_2:CO = 2).

Figure S5 shows the ^{1}H-NMR (500 MHz, $CDCl_3$, RT) and ^{1}H[^{31}P] (500 MHz, $CDCl_3$, RT) spectra for a RHF reaction liquor in the absence of a Fischer-Tropsch catalyst. A solution of Co_2CO_8 and PCy_3 (Co(M):P(L) atomic ratio of 1:1) in *n*-hexane was exposed to RHF conditions for one hour (see caption to Figure S5 for experimental details). In good accordance with the ^{31}P-spectrum (Figure S4), the ^{1}H-spectrum shows a duplet in the hydride region with a chemical shift of δ = –10.9 ppm and a coupling of J_{P-H} = 41 Hz, which can be assigned to the main species [HCo(CO)$_3$(PCy$_3$)] (Figure S3, **iv**). Upon phosphorus decoupling, the duplet in the ^{1}H[^{31}P] spectrum collapses to a singlet. The ^{1}H chemical shift shows good accordance with those reported in literature for similar species.[6a, 8]

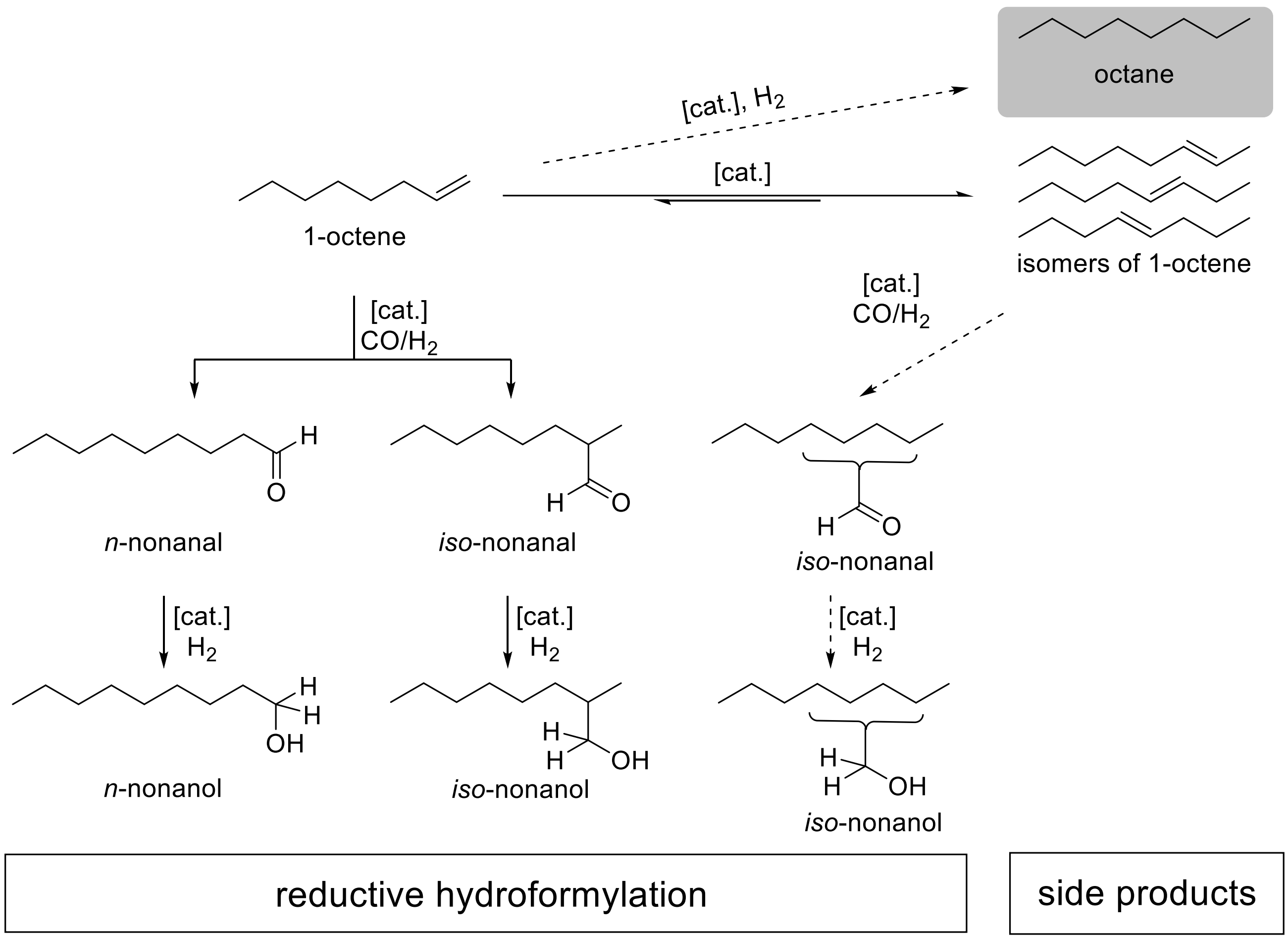

Figure S6: Scheme for the reductive olefin hydroformylation including potential side-reactions, i.e. olefin hydrogenation and double-bond isomerization. *1*-octene is considered in the scheme as a model compound for linear Fischer-Tropsch *1*-olefin products.

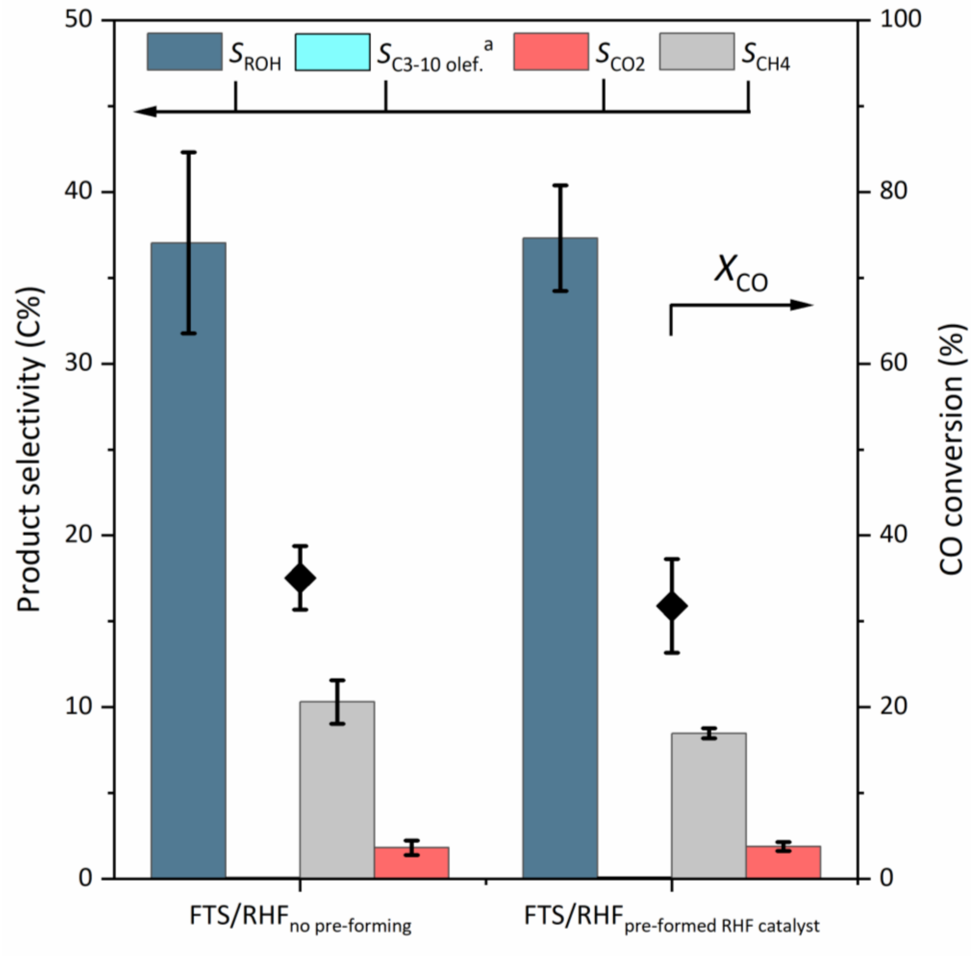

Figure S7: Influence of RHF catalyst pre-forming on the final performance of the tandem Fischer-Tropsch synthesis/reductive olefin hydroformylation syngas conversion process. Product selectivity (bars, left y-axis) and CO conversion (scatter symbols, right y-axis) for slurry-phase tandem FTS/RHF experiments adding FTS and RHF components simultaneously (left) or with pre-forming the RHF catalyst in an initial step for 1 h prior to the addition of the FTS catalyst (right). FTS catalyst: NaPr-CoRu/AOmM; RHF catalyst: Co_2CO_8 + PCy_3 (L:M=1.0 (P/Co, at/at)). Reaction conditions: T = 473 K, P= 120 bar (initial, measured at RT), solvent *2*-methyl pentane, stirring rate 700 rpm, syngas feed H_2:CO = 2. [a]$C_{3\text{-}10}$ olefin selectivity ≤ 0.2 C% in all cases.

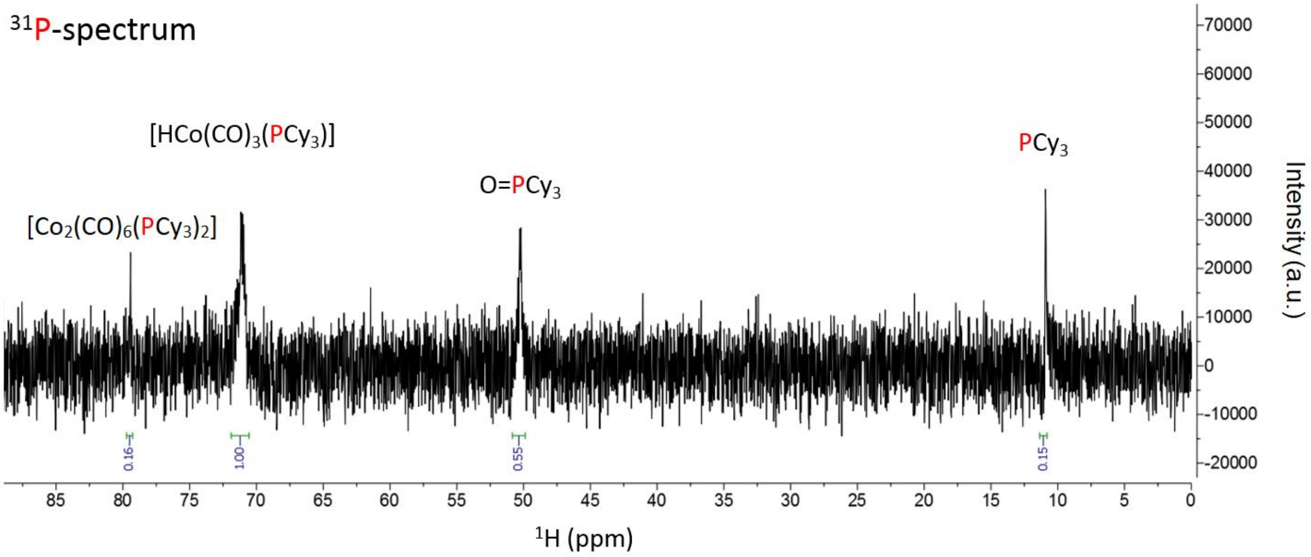

Figure S8: Analysis of molecular RHF catalyst species in tandem FTS/RHF catalytic process. $^{31}P[^{1}H]$ (500 MHz, $CDCl_3$, RT) spectrum of the reaction liquor for a FTS/RHF tandem reaction test.[7] Reaction conditions: *m*(FTS catalyst) = 40.1 mg, *n*($Co_2(CO)_8$) = 140 µmol, *n*(PCy_3) = 140 µmol, V(*n*-hexane) = 1.37 mL, P = 120 bar (measured at RT), H_2:CO= 2, T = 468 K, t = 24 h.

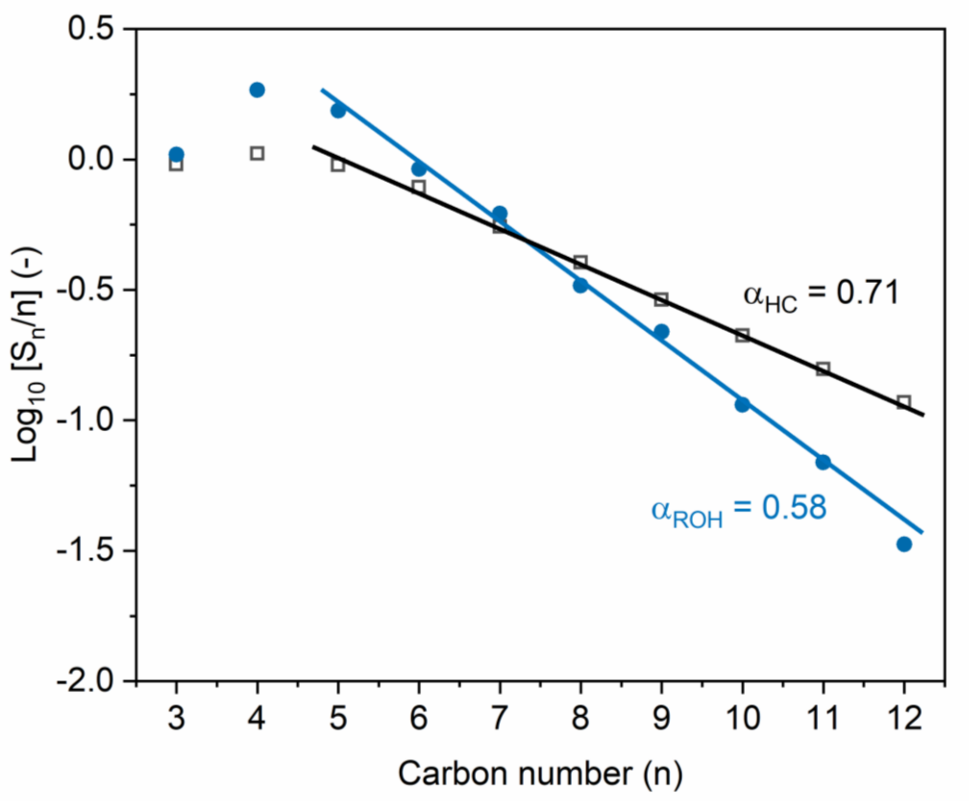

Figure S9: Representative linearized Anderson-Schulz-Flory plot for hydrocarbon (open squares) and alcohol (full circles) products from the slurry-phase FTS/RHF tandem conversion of syngas.

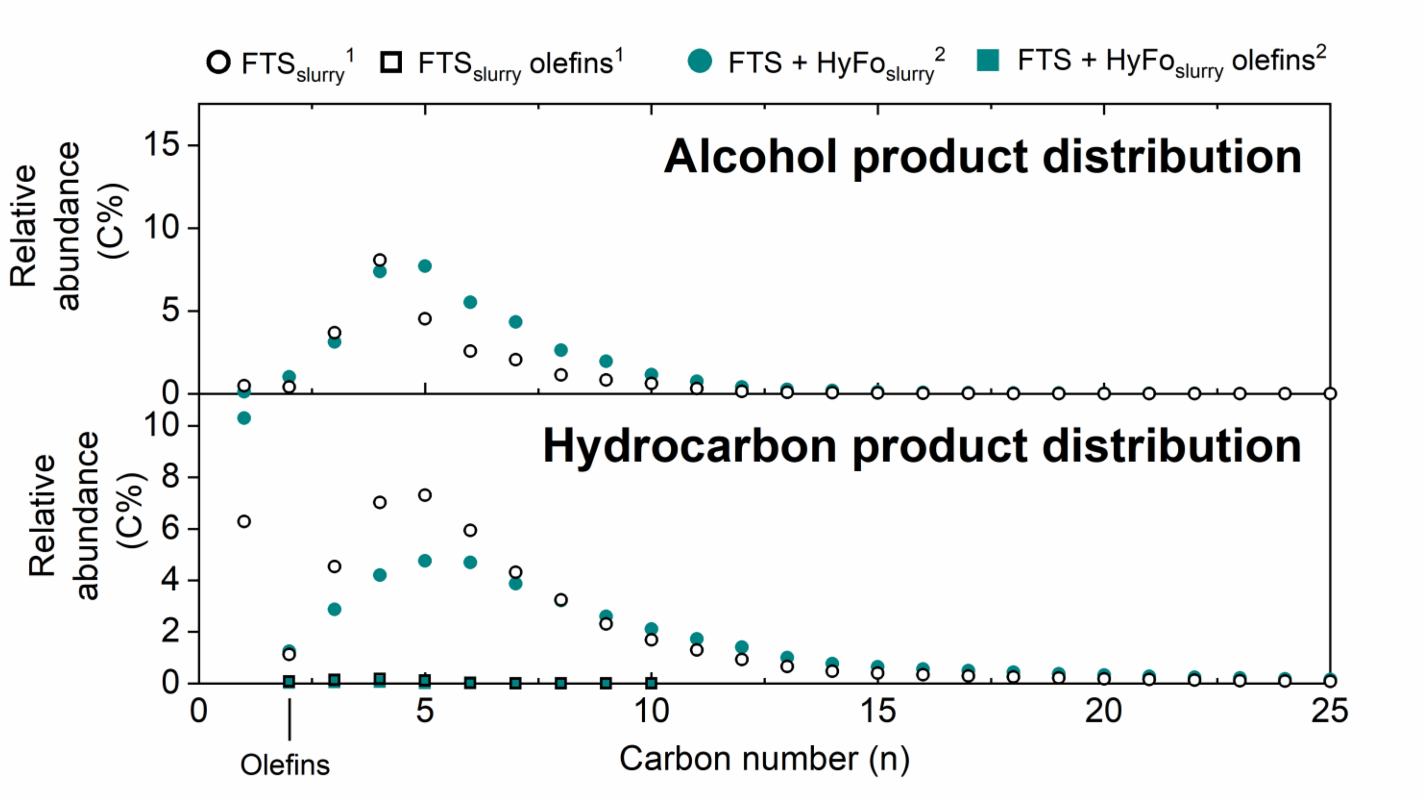

Figure S10: Alcohol and hydrocarbon product distributions, based on head space + offline gas chromatography analyses, obtained for slurry-phase direct syngas conversion via the optimized NaPr-CoRu/AOmM Fischer-Tropsch catalyst (black, open symbols) and the tandem Fischer-Tropsch synthesis/reductive olefin hydroformylation catalyst combination of NaPr-CoRu/AOmM + $Co_2(CO)_8$ + PCy_3 (green, closed symbols). Co_2CO_8 + PCy_3 (L:M=1.0 (P/Co, at/at)). Olefin contribution to alcohols and hydrocarbons shown in square symbols, olefin selectivity for these test was ≤ 0.2 C% T = 473 K, P= 120 bar (initial, measured at RT), solvent *2*-methyl pentane, stirring rate 700 rpm, syngas feed H_2:CO = 2.

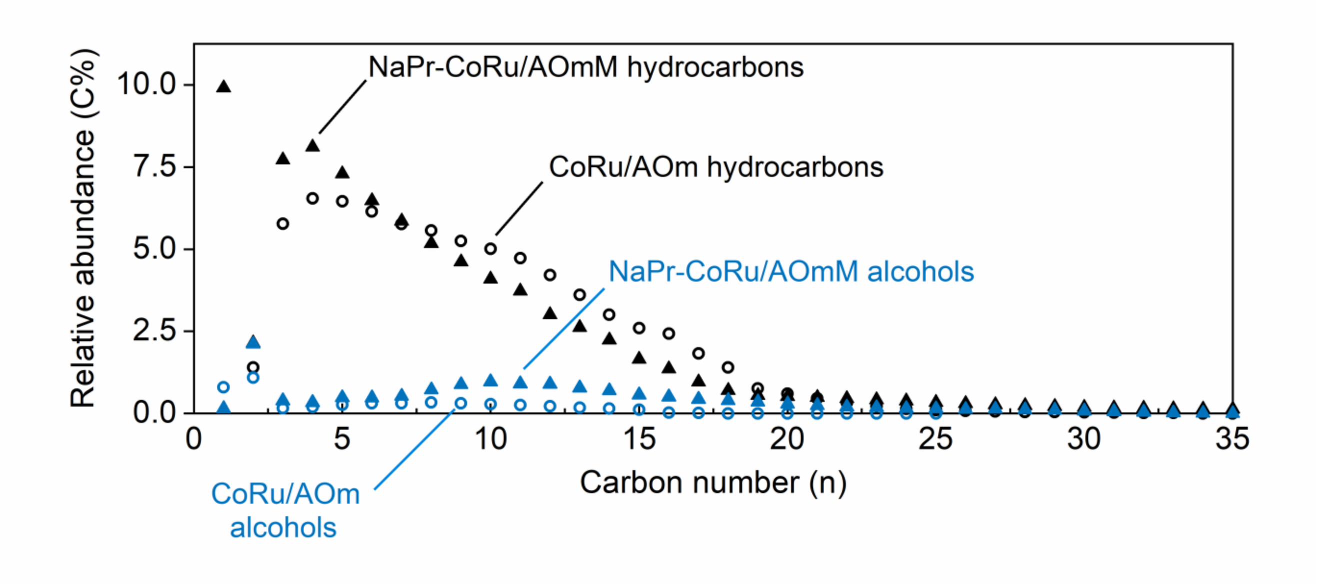

Figure S11: Alcohol and hydrocarbon product distributions, based on integrated online + offline gas chromatography analyses, obtained in the pseudo-steady state (ToS = 166-184 h) for fixed-bed Fischer-Tropsch synthesis tests with cobalt-based catalysts CoRu/AOm and NaPr-CoRu/AOmM. Reaction conditions: T = 473 K, P = 20 bar, H_2:CO = 2, X_{CO}=20 ± 3 %. Only terminal *n*-alcohols (*n:iso*~∞) were detected in these FTS tests.

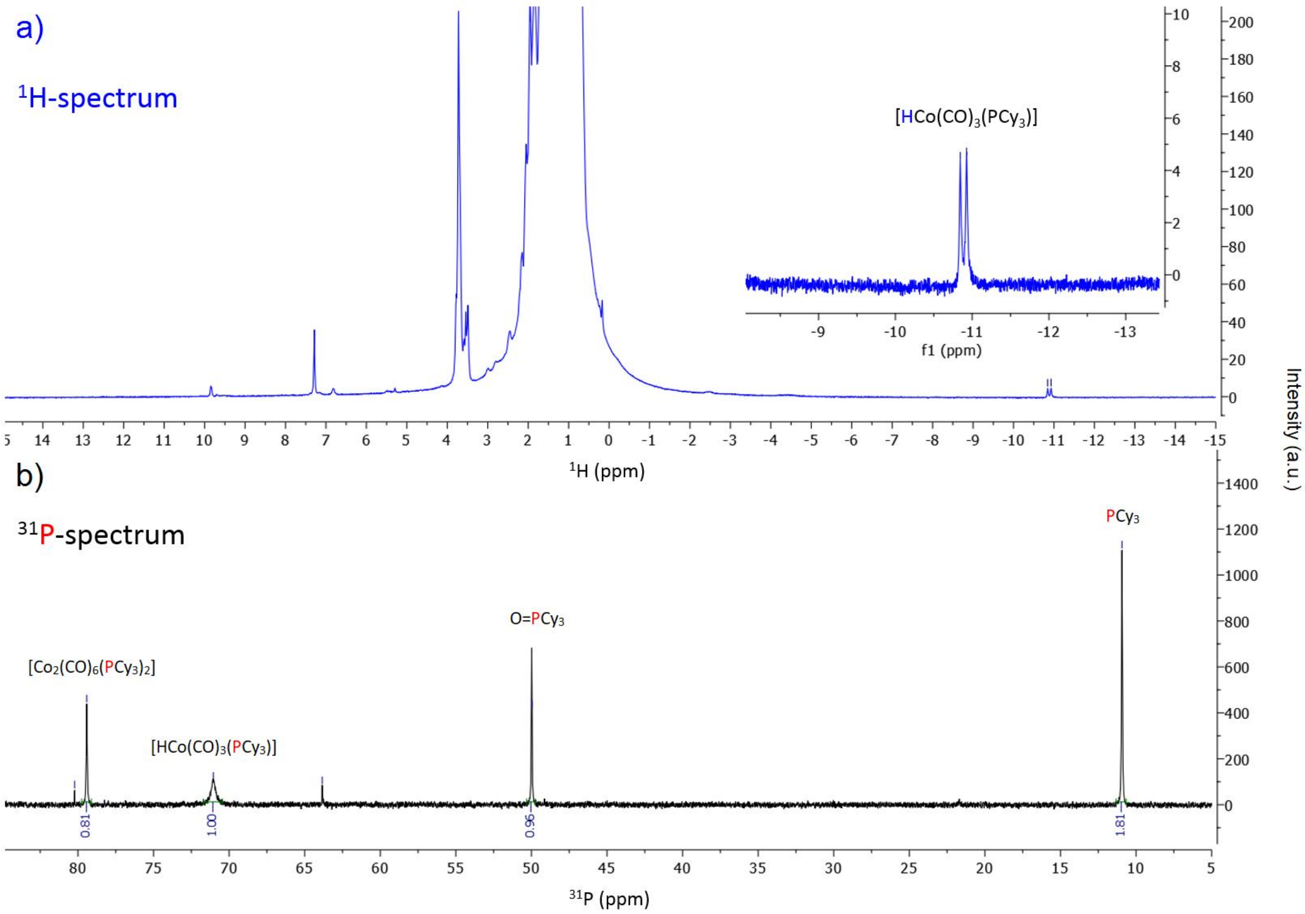

Figure S12: Analysis of molecular RHF catalyst species developed upon interaction of PCy_3 ligand with the solid FTS catalyst under slurry-phase reaction conditions without a specific molecular cobalt source. a) ^{1}H-NMR (500 MHz, $CDCl_3$, RT) spectrum, and b) ^{31}P[^{1}H]-NMR (202 MHz, $CDCl_3$, RT) spectrum of the postreaction liquor of an experiment where PCy_3 was added to a solid FTS catalyst (NaPr-CoRu/AOmM). Reaction conditions: m(FTS catalyst) = 40.1 mg, n(PCy_3) = 140 µmol, V(*n*-hexane) = 1.37 mL, P = 120 bar (measured at RT), H_2:CO=2, T = 468 K, *t* = 24 h.

As shown in Figure S12, following addition of the PCy_3 ligand to a suspension of the solid NaPr-CoRu/AOmM Fischer-Tropsch catalyst and exposure to syngas conversion conditions for 24 h, the ^{1}H-NMR spectrum for the reaction liquor revealed the same hydride species at δ = –10.9 ppm which was identified earlier as [$HCo(CO)_3(PCy_3)$], even though no specific cobalt source had been added for the development of the RHF molecular catalyst. The presence of cobalt phosphorous species is confirmed in the corresponding ^{31}P-NMR spectrum. Besides free phosphorus ligand PCy_3 and oxidized O=PCy_3 in high concentrations, the hydride species [$HCo(CO)_3(PCy_3)$] and the dinuclear species [$Co_2(CO)_6(PCy_3)_2$] can be identified in the ^{31}P-spectrum. Even though at remarkably lower concentrations compared to the case where a specific cobalt carbonyl source is added into the reaction medium, the detection of these species, which take part in the RHF pre-catalyst equilibria (Figure S3), suggests their development via ligation of a small fraction of cobalt species leached from the surface of the solid FTS catalyst.

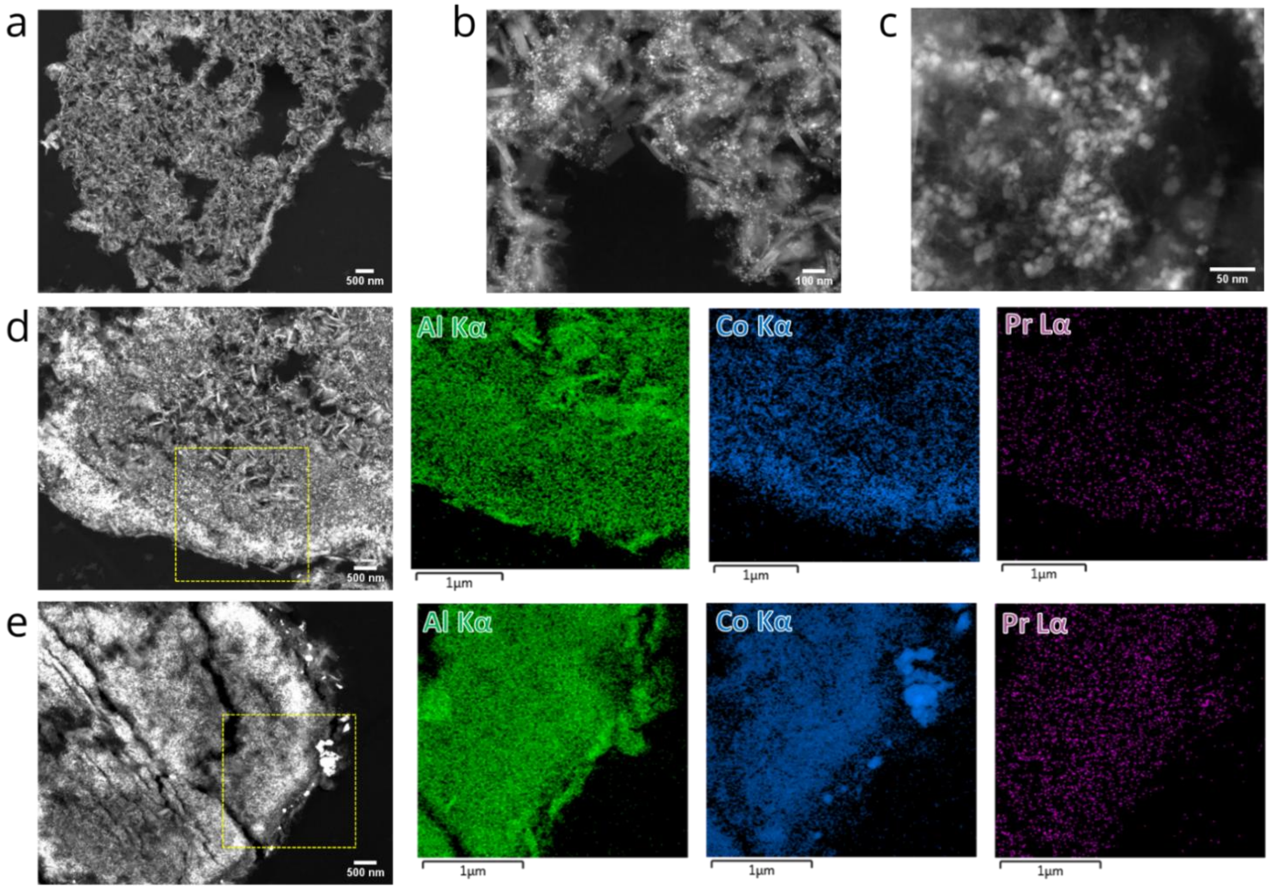

Figure S13: Nanostructural characterization of an olefin-selective cobalt Fischer-Tropsch catalyst after application in the tandem FT-RHF process. a-c) Representative HAADF-STEM micrographs, at different magnifications, and d-f) HAADF-STEM micrographs alongside EDS compositional maps (from the areas framed on the micrographs), for ca. 150 nm thin cross-sections of the NaPr-CoRu/AOmM Fischer-Tropsch solid catalyst recovered after a FTS/RHF test. Reaction conditions: FTS catalyst NaPr-CoRu/AOmM; RHF catalyst: Co_2CO_8 + $P(Cy)_3$ (L:M=1.0 (P/Co (at/at), T = 473 K, P= 120 bar (initial, measured at RT), stirring rate 700 rpm, syngas feed H_2:CO = 2, *2*-methyl pentane as solvent, t=24 h.

Electron microscopy was applied on the NaPr-CoRu/AOmM Fischer-Tropsch catalyst to assess any (nano)structural modifications brought about by its exposure to reaction conditions in the slurry-phase FTS/RHF process. The results are summarized in Figure S13. The catalyst particles preserved their intraparticle macroporosity (see panel a). Observation at high magnification revealed that the size and uniform spatial distribution of the Co nanoparticles within the mesoporous domains of the Al_2O_3 carrier had been preserved. EDS nanospectroscopy compositional maps revealed, however, a certain enrichment in cobalt close to the outer rim of the catalyst particles, alongside, few (scarce) large Co aggregates (see panels d and e), which are compatible with the deposition of cobalt from the molecular Co carbonyl catalyst and/or its decomposition/precipitation upon isolation and drying of the solid catalyst from the reaction liquors. Extensive EDS analyses did not reveal any relevant phosphorous content, reinforcing the stability of the molecular catalyst which remains by and large soluble after catalysis and it is therefore removed during the centrifugation workup applied to isolate the solid FT catalyst from the reaction liquors.

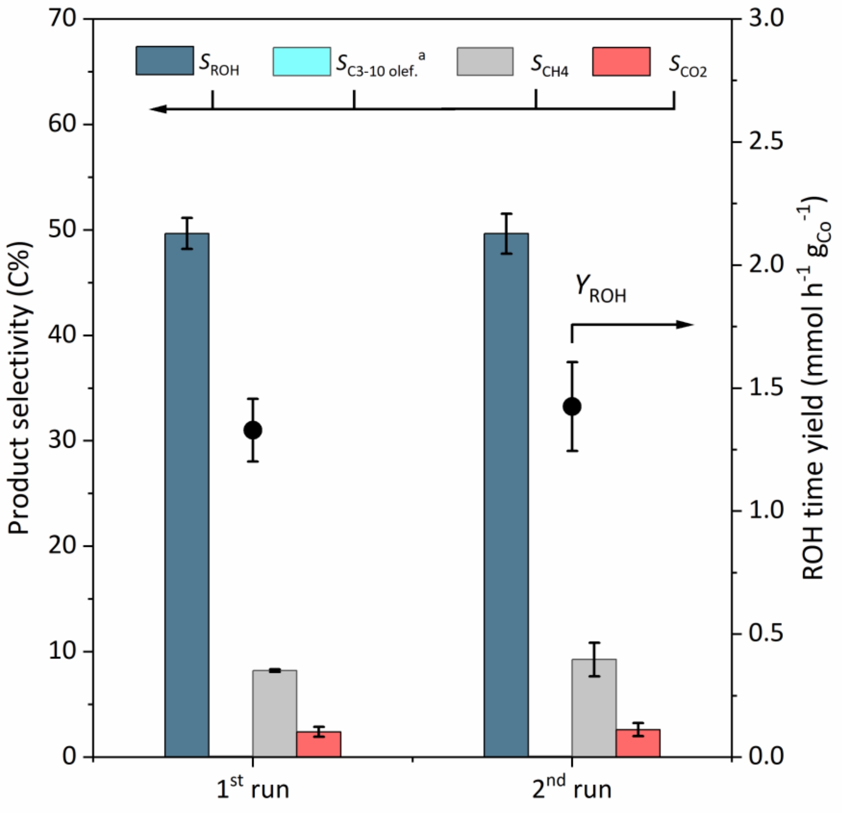

Figure S14: FTS catalyst recycling. Catalyst performance for two consecutive FTS/RHF tandem reaction runs. The reaction liquors were hot filtered off the reactor following the first run, under a protective Ar atmosphere. The solid FTS catalyst was recovered and applied in a second reaction run without any rejuvenation/regeneration. FTS catalyst: NaPr-CoRu/AOmM; RHF catalyst: Co_2CO_8 + PCy_3 (L:M=1.0 (P/Co, at/at)). Reaction conditions: T = 473 K, P= 120 bar (initial, measured at RT), solvent *2*-methyl pentane, stirring rate 700 rpm, syngas feed H_2:CO = 2. [a]$C_{3\text{-}10}$ olefin selectivity $\leq$ 0.2 C% in all cases. Activity is normalized per unit metal mass (metal-specific time yield) to account for the fact that the solid FTS catalyst could not be recovered quantitatively from the cake formed on the filter during hot filtration after the first run.

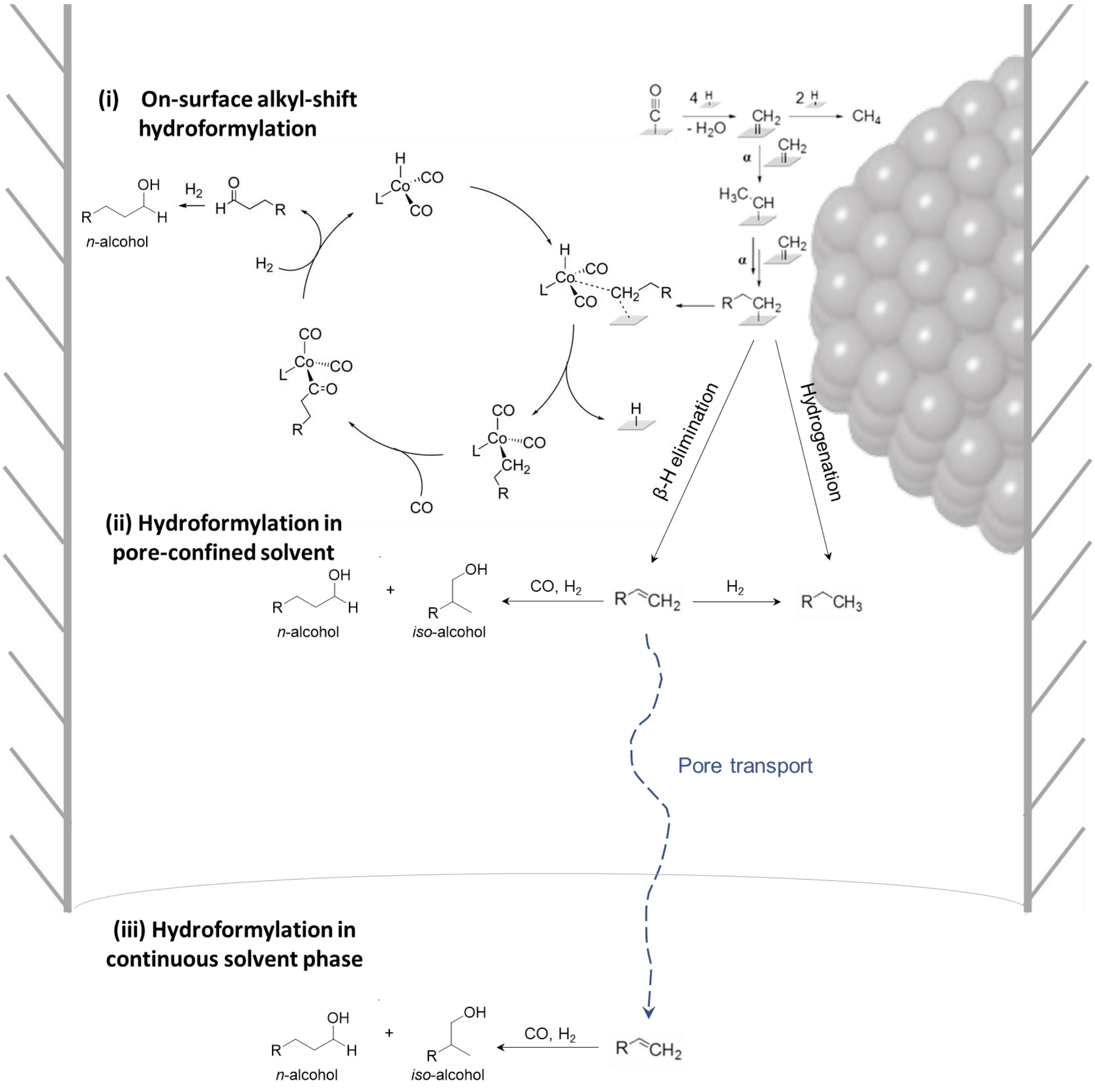

Figure S15: Three mechanistic proposals for the reductive hydroformylation of α-olefins produced *in situ* from syngas on a FTS solid catalyst: (i) alkyl-shift mechanism wherein the molecular cobalt carbonyl RHF catalyst intercepts α-C-bonded growing hydrocarbon chains from the surface of the Co nanoparticles on the FTS catalyst, prior to their desorption; (ii) desorption of α-olefin primary products from the surface of the Co nanoparticles followed by their interception by the molecular cobalt carbonyl RHF catalyst immediately after desorption, within the pore-occluded liquid-phase; (iii) desorption of α-olefin primary products from the surface of the Co nanoparticles followed by pore transport, egression from the porosity of the FTS catalyst and conversion by the molecular cobalt carbonyl RHF catalyst in the extra-pore (continuous) liquid phase. For simplicity the scheme does not contemplate side reactions initiated via double-bond isomerization of FTS *1*-olefins, and the full hydroformylation mechanism proposal is only showed for the case of the on-surface alkyl-shift.

a

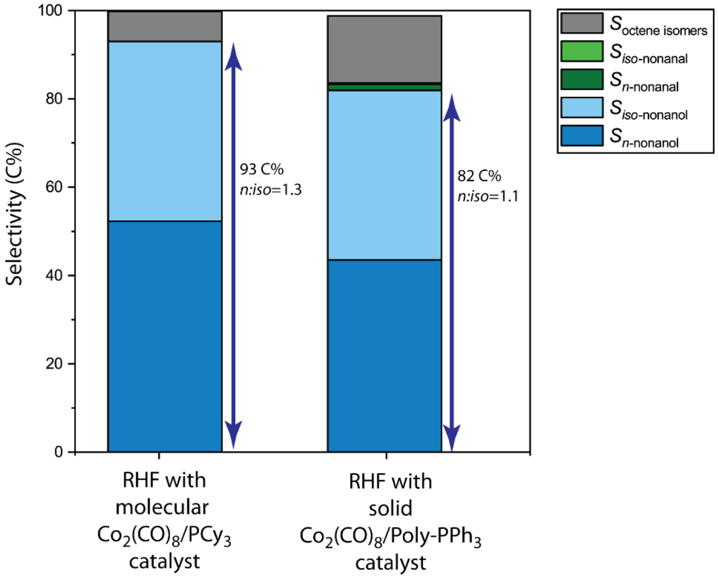

b

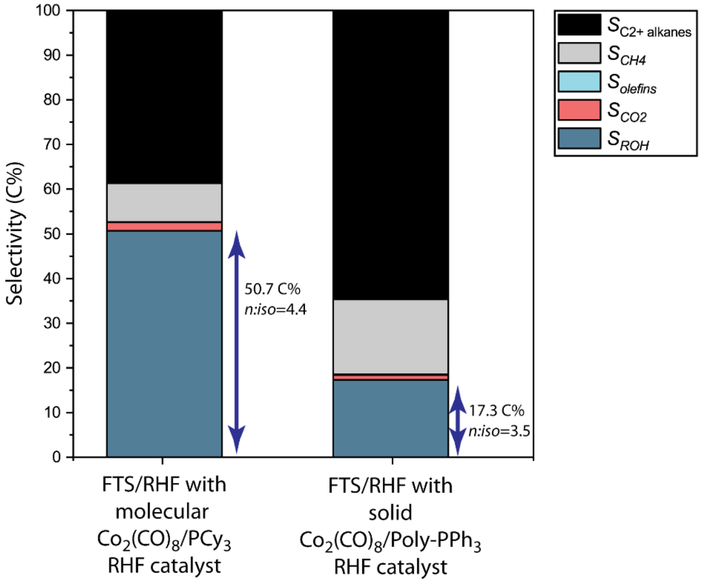

Figure S16: a) Comparison of the performance of free molecular $Co_2(CO)_8/PCy_3$ and solid polymer-tethered $Co_2(CO)_8$/Poly-PPh_3 catalysts in the slurry-phase reductive hydroformylation of *1*-octene model FTS *1*-olefin. Reaction conditions: n(Co) = 140 µmol, n(P) = 140 µmol, L:M=1, n(*1*-octene) = 9.3 mmol, V(*n*-hexane) = 2.75 mL, T = 468 K, H_2:CO=2.0, t = 3 h. *1*-octene conversion was >99% for both reactions. b) Comparison of the performance of free molecular $Co_2(CO)_8/PCy_3$ and solid polymer-tethered $Co_2(CO)_8$/Poly-PPh_3 RHF catalysts in the direct syngas conversion to higher alcohols by the tandem FTS/RHF process. FTS catalyst: NaPr-CoRu/AOmM; RHF catalyst: L:M=1; $Co_{FTS}:Co_{RHF}$ = 170:70. Blue arrows highlight the total alcohol selectivity on the bar plot and the corresponding labels include the alcohol *n*:*iso* molar ratio. Reaction conditions: T = 473 K, P = 120 bar (initial, measured at RT), stirring rate 700 rpm, syngas feed H_2:CO = 2.

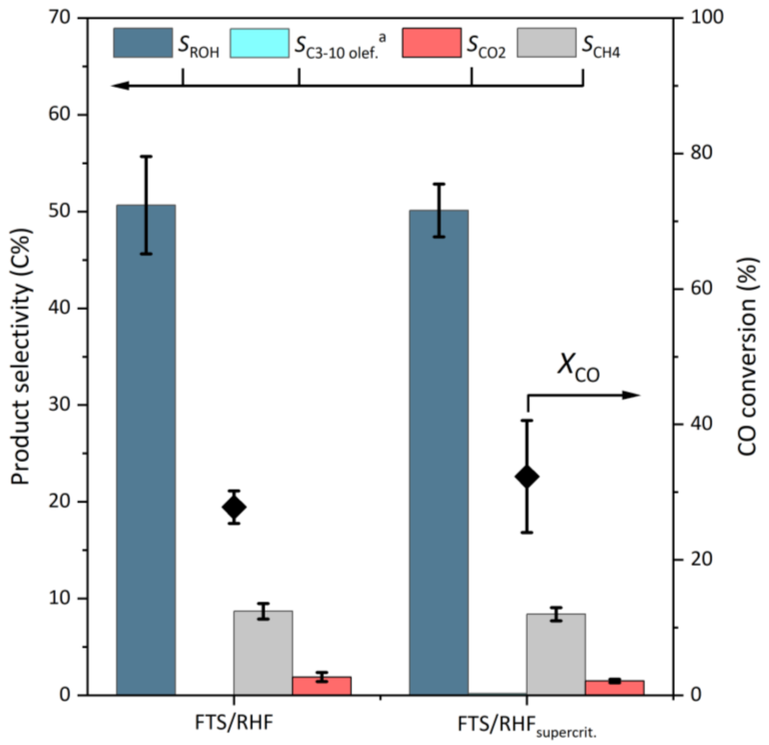

Figure S17: Impact of liquid vs supercritical reaction medium in the direct syngas conversion to higher alcohols via the tandem slurry-phase Fischer-Tropsch synthesis/reductive olefin hydroformylation. Product selectivity (left y-axis) and CO conversion (right y-axis) for standard liquid slurry-phase tandem FTS/RHF in 2-methyl-pentane solvent, which is liquid under the applied reaction conditions (left) and the supercritical-phase tandem FTS/RHF in 2-methyl-butane solvent, which is in supercritical state under the reaction conditions (right). FTS catalyst: NaPr-CoRu/AOmM; RHF catalyst: Co_2CO_8 + PCy_3 (L:M = 1.0 (P/Co, at/at)), Co_{FTS}:Co_{RHF} = 170:70. Reaction conditions: T = 473 K, P = 120 bar (initial, measured at RT), stirring rate 700 rpm, syngas feed H_2:CO = 2. [a]C_{3-10} olefin selectivity ≤ 0.2 C% in all cases.

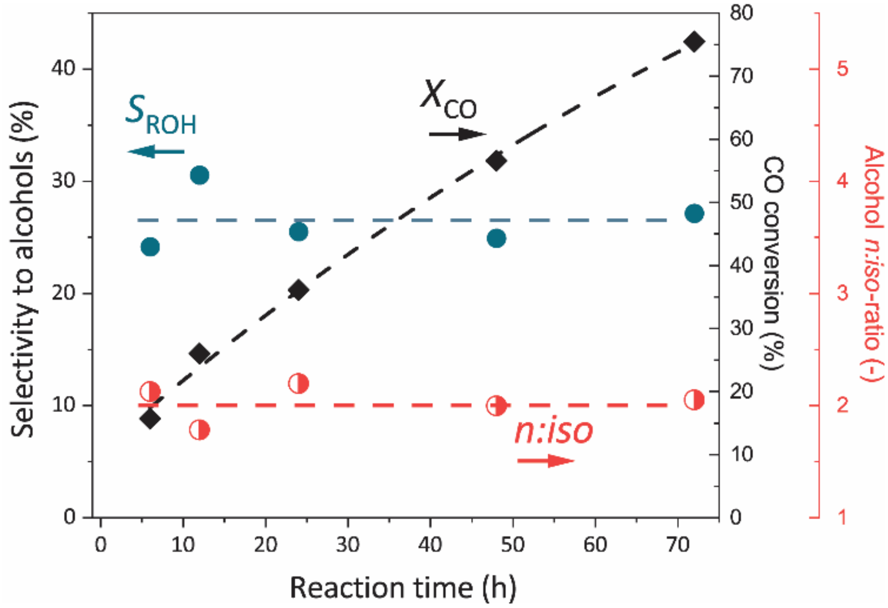

Figure S18: Time-resolved evolution of CO conversion and alcohol product (regio)selectivity during slurry-phase syngas conversion reaction tests with catalyst the NaPr-CoRu/AOmM solid FTS catalyst alone. Reaction conditions: T = 473 K, P= 120 bar (initial, measured at RT), stirring rate 700 rpm, syngas feed H_2:CO = 2, 2-methyl pentane as solvent. Dotted lines are included as guides to the eye.

As shown in Figure 4 (main text), time-resolved monitoring of the bifunctional process showed the CO conversion to increase essentially linearly with time to reach 70.1% after 72 h. A comparatively similar trend was observed with the FTS alone (Figure S18 above), discarding any noticeable deactivation of the latter due to either the concomitant presence of the RHF catalyst or the increase in water content in the reaction medium at increasingly higher syngas conversion levels. Moreover, alcohol selectivity scaled steeply with time up to ca. 20 h, suggesting a transient induction period for RHF activity (Figure 4), to level off to ca. 50-53 C% at CO conversion levels in the range of 30-50%, and slightly decrease to just above 45 C% when the CO conversion was further increased to ~70%. It is surmised that this slight decrease in alcohol selectivity is associated to the drop in P_{CO} to 12.1 bar observed at this syngas conversion level in the batch reaction system, i.e. lower than the critical P_{CO} below which the molecular RHF catalyst offers suboptimal performance. Remarkably, a gradual and marked increase in *n*:*iso* ratio was observed for the alcohol products (from ca. 1.5 to >5.0) on increasing CO conversion up to >50% with the tandem catalysis system. (Figure 4). Meanwhile, identical experiments with the solid FTS as the sole catalyst (Figure S18) showed constant alcohol selectivity of 27.3 ±3.2 C% and alcohol *n*:*iso* ratio of ca. 2 over the same syngas conversion span. In view of the pronounced effect which the L:M ratio has on the regioselectivity of the RHF reaction (Table S3), we surmise that a progressive increment in the effective ligand-to-cobalt ratio might underlie the trend observed for the higher alcohol regioselectivity in the tandem process, possibly as a result of a partial depletion of the molecular catalyst in Co upon the establishment of dissolution/deposition equilibria with the cobalt agglomerates on the FTS catalyst.

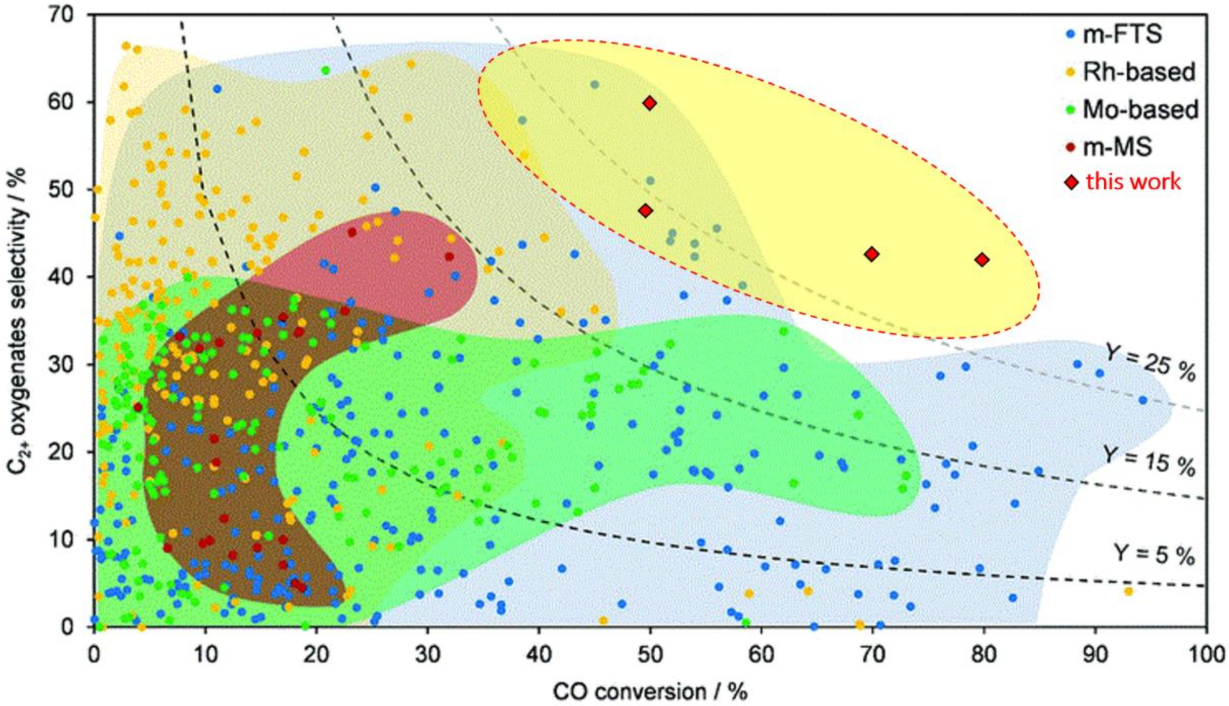

Figure S19: Comparison of the performance of the newly developed tandem FTS/RHF process to the state-of-the-art in the direct conversion of syngas to higher alcohols. The plot shows the selectivity to higher (C_{2+}) oxygenates as a function of CO conversion for research high-alcohol synthesis (HAS) catalysts within four main families, i.e., modified FTS catalysts (m-FTS, blue), Rh-based catalysts (Rh-based, orange), Mo-based catalysts (Mo-based, green) and modified MS catalysts (m-MS, dark red) in an overview graph, as surveyed by Luk et al. Adapted from [9]. Selected performance data for the herein reported tandem FTS/RHF process have been added on the plot (red diamonds).

4. Supporting Tables

Table S1: Reaction rate and product selectivities for the gas-phase Fischer-Tropsch synthesis (FTS) reaction (fixed-bed reactor) with CoRu-based FTS catalysts. Reaction conditions: T = 473 K, P = 20 bar, H_2:CO = 2, CO conversion = 20 ± 3%.

Entry	Catalyst	CTY[a] [mmolCO$g_{Co}^{-1}h^{-1}$]	$S(CO_2)$ [C%]	$S(CH_4)$ [C%]	$S(C_{5+})$ [C%]	$S(C_{3-10}^{=})$[b] [C%]	α_{HC}[c] [-]	S_{MeOH} [C%]	$S(C_{2+ROH})$[d] [C%]	α_{ROH}[e] [-]
1.1	CoRu/AOm	149.9	0.4	14.2	70.3	16.3	0.79	0.6	5.0	0.70
1.2	NaPr-CoRu/AOmM	46.9	0.9	9.9	68.6	31.8	0.77	0.1	14.4	0.74

[a] Cobalt-time-yield. [b]Selectivity to C_{3-10} olefins. [c]Hydrocarbon chain-growth probability. [d]Selectivity to C_{2+} alcohols. [e]Alcohol chain-growth probability.

Table S2: Performance screening for a $Co_2(CO)_8$ + PCy_3 catalyst for RHF of *1*-octene as model compound for FTS *1*-olefins. Influence of syngas pressure.

Entry	P[a] [bar]	P_{CO}[a] [bar]	$X_{1\text{-octene}}$ [%]	$S_{alcohols}$[b] [%]	$S_{aldehydes}$[b] [%]	S_{octane} [%]	$S_{octene\ isomers}$ [%]	*n:iso*[c]	$TON_{alc.}$[d]
2.1	120	40	> 99	93	<1	<1	7	1.3	62
2.2	90	30	> 99	74	17	<1	9	1.6	49
2.3	60	20	> 99	64	17	<1	19	1.5	42
2.4	30	10	> 99	16	4	9	68	1.5	11

Reaction conditions: *n*(Co) = 140 µmol, *n*(PCy_3) = 140 µmol, L:M=1:1, *n*(*1*-octene) = 9.3 mmol, V(*n*-hexane) = 2.75 mL, T = 468 K, H_2:CO = 2.0, *t* = 3 h. *X* = conversion, *S* = selectivity, M = metal, L = ligand. [a] Initial, measured at RT. [b] Lumped selectivity to *n*- and *iso*-products. [c] linear-to-branched ratio for alcohol products. [d] *Turnover number* for alcohol production.

The performance of the $Co_2(CO)_8$ + PCy_3 catalyst was assessed for the reductive hydroformylation of *1*-octene, as a model compound for mid-chain Fischer-Tropsch synthesis α-olefins. The reaction temperature of 468 K was selected for compatibility with the operation temperature typical for the FTS. As shown in Table S2, the catalyst showed high activity for the conversion of *1*-octene, and full olefin conversion was achieved within 3 hours of reaction in all cases. Under a syngas pressure of 120 bar (corresponding to a CO partial pressure P_{CO} of 40 bar) a high selectivity of 93% to alcohols was achieved. Under these conditions, the selectivity towards the full olefin hydrogenation product (*1*-octane) was very low (<1%). The suppression of this side-reaction, which is commonly observed in olefin reductive hydroformylation reactions,[10] is particularly relevant in the contest of the FTS/RHF tandem process. Decreasing the syngas pressure stepwise to 90, 60 and 30 bar (corresponding to P_{CO} in the range of 30-10 bar) led to a decrease in alcohol selectivity and an increase in the selectivity to internal *1*-octene isomer olefins. Below 60 bar of total pressure, the hydrogenation of the starting olefin is enhanced significantly. The deterioration of the product selectivity was particularly notorious when the CO partial pressure was decreased to 10 bar, where the selectivity to *n*-octane reached 9% and that to internal olefin isomers approached 70%. The syngas pressure had little influence on the regioselectivity of the alcohol products, for which the *n:iso* ratio remained within 1.3-1.6.

It is known that cobalt-based catalysts generally need comparatively high partial CO pressures than rhodium-based catalysts in hydroformylation reactions.[11] Given that the Fischer-Tropsch synthesis is not substantially influenced by the total reaction pressure, a total initial syngas pressure of 120 bar was selected for the tandem FTS/RHF experiments.

Table S3: Performance screening for a $Co_2(CO)_8$ + PCy_3 catalyst for the RHF of *1*-octene as model compound for FTS *1*-olefins. Influence of the ligand-to-metal ratio and catalyst concentration.

Entry	L:M[a] ratio	c(Co)[b] [mol%]	$X_{1\text{-octene}}$ [%]	$S_{alcohols}$[c] [%]	$S_{aldehydes}$[c] [%]	S_{octane} [%]	$S_{octene\ isomers}$ [%]	*n:iso*[d]	$TON_{alc.}$[e]
3.1	1:1	1.5	>99	92	<1	1	6	1.4	62
3.2	2:1	1.5	>99	90	<1	<1	9	3.0	60
3.3	4:1	1.5	99	83	1	3	12	4.4	56
3.4	10:1	1.5	99	61	2	17	19	12	41
3.5	1:1	0.75	>99	88	<1	<1	11	1.1	117
3.6	1:1	0.38	>99	87	<1	<1	12	1.0	228
3.7	1:1	0.19	>99	84	3	<1	12	1.1	442

Reaction conditions: n(*1*-octene) = 9.3 mmol, V(*iso*-hexane) = 2.75 mL, H_2:CO=2.0, P = 120 bar (initial, measured at RT), T = 468 K, t = 3 h. X = conversion, S = selectivity, M = metal, L = ligand. [a] Ligand-to-metal ratio, expressed as P/Co (at/at). [b] Cobalt concentration in the reaction medium relative to the initial *1*-olefin substrate concentration. [c] Lumped selectivity to *n*- and *iso*-products. [d] linear-to-branched ratio for alcohol products. [e] *Turnover number* for alcohol production.

Increasing the ligand-to-metal ratio from 1:1 to 10:1 resulted in a progressive and marked increase in the linear-to-branched ratio for the alcohol products, from 1.4 up to 12, indicative for a progressively enhanced anti-Markovnikov regioselectivity and/or inhibited double-bond isomerization reactivity prior to olefin oxo-functionalization (Table S3, entries 3.1-3.4). With a ligand-to-metal ratio of 10:1, *1*-nonanol accounts for more than 90% of the alcohols produced. Together with the increase in alcohol regioselectivity, an increase in the undesired direct olefin hydrogenation, alongside a reduced activity for the transformation of internal olefins, are observed, which lower the overall selectivity to alcohols. Literature describes a monocoordinated cobalt complex as the active catalyst for the cobalt-catalyzed hydroformylation reaction using monodentate trialkylphosphine compounds as ligands.[6a, 7] With higher ligand contents, the development of cobalt molecular species with higher ligand coordination degrees (higher substituted species) are considered to be sterically more demanding, which might induce higher regioselectivity, but not as active as the mono-substituted cobalt complex.[6a] Moreover, the application of a ligand excess might lead to non-coordinated (free) phosphine species. Given that the Fischer-Tropsch catalyst is a well-defined metal surface, interaction between free phosphines and the FTS catalyst surface should be minimized. In view of the higher overall selectivity to alcohols and the expected benefits of a minimum concentration of free ligands in the medium, a ligand-to-metal ratio of 1:1 was selected for the RHF catalyst in FTS/RHF tandem reactions.

As full *1*-octene conversion was achieved during previous experiments, the catalyst concentration in the reactor was lowered in subsequent experiments (Table S3, entries 3.5-3.7). Even upon lowering the catalyst loading from 1.5 mol% (Entry 3.1) to 0.19 mol% (Entry 3.7), full *1*-octene conversion was achieved, alongside a significant increase in the *Turnover Number* (TON) from 63 to 389, showing that the RHF catalyst is much more active than observed during the initial experiments with higher catalyst concentrations.

Table S4: Cobalt concentration as determined by inductively-coupled plasma mass-spectrometry (ICP-MS) in the liquors recovered from slurry-phase catalytic tests after 24 hours of reaction time. The Fischer-Tropsch synthesis (FTS) catalyst was NaPr-CoRu/AOmM in all cases. PCy_3: tricyclohexyl phosphine.

Entry	Catalyst/s	[Co] [μg/mL]
4.1	FTS + Co_2CO_8/PCy_3	685
4.2	FTS + PCy_3	51
4.3	FTS	7

Table S5: Investigation of product regioselectivity for Fischer-Tropsch synthesis (FTS)-derived and non-FTS tracer α-olefin educts in the tandem reaction FTS/RHF (reductive hydroformylation). The intrinsic reactivity of the molecular RHF catalyst has been assessed with *5*-methyl-*1*-hexene as model olefin educt. The latter has been additionally applied as a non-FTS (branched backbone) α-olefin tracer olefin in tandem reactions.[1] Molar ratio of *1*-pentanol to *2*-methyl-*1*-pentanol for tests with *5*-methyl-*1*-hexene as educt.[2] Molar ratio of *5*-methyl-*1*-hexanol to 2,4-dimethyl-*1*-pentanol, both originating from the *5*-methyl-*1*-hexene non-FTS tracer olefin. Reaction conditions: 473 K, P= 120 bar (at RT), H_2:CO = 2, t = 24 h, FTS catalyst: NaPr-CoRu/AOmM; RHF catalyst: Co_2CO_8 + PCy_3 (L:M = 1.0 (P/Co, at/at)), Co_{FTS}:Co_{RHF} = 170:70 for entry 5.5. The conversion of the model olefin educts was 84-100%. For tests with liquid olefin educt, hydrogenation product selectivity ≤ 7% in all cases. [3]The Co-free FTS support refers to NaPr/AOmM, i.e. a solid synthesized to have the composition of the FTS catalyst without the active CoRu metals, submitted to the same activation treatment, i.e. reduction in H_2 flow.

Entry	Catalyst/s	Liquid model/tracer olefin reactant	*n*:*iso* ROH from endogenous olefin[1]	*n*:*iso* ROH from model/tracer olefin[2]
5.1	RHF	*5*-methyl-*1*-hexene	---	3.1
5.2	RHF + Co-free FTS support[3]	---	No syngas conversion activity detected	
5.3	RHF + Co-free FTS support[3]	*5*-methyl-*1*-hexene	---	3.7
5.4	FTS	---	2.2	-
5.5	FTS + RHF	*5*-methyl-*1*-hexene (tracer non-FTS α-olefin)	3.0	2.3

The set of experiments summarized in Table S6 was designed to assess whether endogenous α-olefins, i.e. those originating from the FTS conversion of syngas, and an exogenous tracer α-olefin (herein *5*-methyl-*1*-hexene), which is not produced on the surface of the solid FTS catalyst, follow the same or different reductive hydroformylation pathways in the tandem FTS/RHF process. The alcohol product *n*:*iso* ratio was selected as a proxy for the RHF mechanism. Applied on the *5*-methyl-*1*-hexene α-olefin as neat educt, the molecular RHF catalyst ($Co_2(CO)_8$+PCy_3) led to an *n*:*iso* ratio in the resulting alcohol products of 3.1, indicating an intrinsic preference for an anti-Markovnikov reaction regioselectivity. This regioselectivity was maintained when the reaction was performed in the presence of the metal-free, therefore FTS-inactive, porous support material which is part of the composition of the FTS catalyst, suspended in the liquid reaction medium. In the tandem FTS/RHF syngas conversion process, alcohol products originating from the tracer non-FTS α-olefin showed an *n:iso* ratio of 2.3, slightly lower than for alcohols which could be produced as either primary FTS products or as a result of the RHF of FTS olefin intermediate products, which showed an *n*:*iso* ratio of 3.0. This difference in alcohol regio-selectivity can be explained in full by considering the contribution of strictly linear FTS primary products to the latter lump, therefore suggesting that both endogenous (desorbed from the FTS catalyst) α-olefin and the exogeneous tracer α-olefin compounds undergo a mechanistically similar RHF on the molecular catalyst.

Table S6: CO conversion (X_{CO}), and product selectivities for slurry-phase tandem FTS/RHF syngas conversion tests by integration of NaPr-CoRu/AOmM as the Fischer-Tropsch synthesis catalyst and Co_2CO_8 with PCy_3 ligand (at a L:M, i.e. P/Co (at/at), ratio of 1) as the reductive hydroformylation catalyst. Co_{FTS}:Co_{RHF} = 170:70. General reaction conditions: T = 473 K, P = 120 bar (initial, measured at RT), stirring rate of 700 rpm, H_2:CO = 2 (unless stated otherwise), *2*-methyl pentane as solvent.

Entry	nCo_{FTS} [mmol]	t [h]	X_{CO} [%]	$S(CO_2)$ [C%]	$S(CH_4)$ [C%]	$S(C_{5+})$ [C%]	α_{HC}[a] [C%]	$S_{olef.}$ [C%]	S_{ROH} [C%]	$S_{C5+\ ROH}$ [C%]	ROH[b] *n:iso* [-]	α_{ROH}[c] [-]
6.1	0.18	1	1.6	2.7	10.9	61.4	0.67	0.2	24.4	10.3	2.7	0.54
6.2	0.18	2	2.8	2.4	6.6	62.6	0.78	0.2	30.2	17.1	1.5	0.56
6.3	0.18	4	4.9	1.1	8.2	64.6	0.71	0.1	35.8	20.6	2.1	0.59
6.4	0.18	10	14.0	1.4	10.5	60.0	0.71	0.1	39.9	23.0	2.9	0.59
6.5	0.18	16	20.8	1.2	9.8	58.4	0.70	0.1	52.7	30.6	3.4	0.57
6.6	0.18	33	39.2	2.3	9.5	61.9	0.75	0.1	51.5	32.0	4.0	0.59
6.7	0.18	48	47.9	2.7	8.7	57.5	0.72	<0.1	50.4	31.0	5.1	0.61
6.8	0.18	72	70.1	3.4	8.6	57.7	0.73	0.1	44.7	26.6	5.2	0.59
6.9	0.36	36	87.4	5.0	11.5	55.1	0.68	<0.1	37.5	24.1	6.2	0.70
6.10	0.27	48	80.4	4.8	8.9	54.3	0.71	0.1	42.7	27.2	6.1	0.70
6.11[d]	0.36	72	52.3	8.5	7.0	56.9	0.72	<0.1	60.1	34.4	3.9	0.60

[a]Hydrocarbon chain-growth probability. [b]linear-to-*iso* alcohol ratio [c]Alcohol chain-growth probability [d]H_2:CO = 1.

5. References

[1] R. C. Reuel, C. H. Bartholomew, *J. Catal.* **1984**, *85*, 78-88.

[2] F. Meyer, S. Beucher, *J. Vis. Commun. Image Represent* **1990**, *1*, 21-46.

[3] G. Borgefors, *Comput. Vis. Image Underst.* **1996**, *64*, 368-376.

[4] N. Iversen, B. B. Jørgensen, *Geochim. Cosmochim. Acta* **1993**, *57*, 571-578.

[5] M. I. Fadlalla, S. G. Babu, T. M. Nyathi, C. J. Weststrate, N. Fischer, J. W. H. Niemantsverdriet, M. Claeys, *ACS Catal.* **2020**, *10*, 14661-14677.

[6] a) C. Dwyer, H. Assumption, J. Coetzee, C. Crause, L. Damoense, M. Kirk, *Coord. Chem. Rev.* **2004**, *248*, 653-669; b) M. Absi-Halabi, J. D. Atwood, N. P. Forbus, T. L. Brown, *J. Am. Chem. Soc.* **1980**, *102*, 6248-6254.

[7] C. D. Wood, P. E. Garrou, *Organometallics* **1984**, *3*, 170-174.

[8] K. W. Kramarz, R. J. Klingler, D. E. Fremgen, J. W. Rathke, *Catal. Today* **1999**, *49*, 339-352.

[9] H. T. Luk, C. Mondelli, D. C. Ferré, J. A. Stewart, J. Pérez-Ramírez, *Chem. Soc. Rev.* **2017**, *46*, 1358–1426.

[10] H. M. Torres Galvis, K. P. de Jong, *ACS Catal.* **2013**, *3*, 2130-2149.

[11] R. Franke, D. Selent, A. Börner, *Chem. Rev.* **2012**, *112*, 5675-5732.
